# Distinct Molecular Biomechanical Mechanisms Inhibit Endosperm Cell‐Wall Weakening and Seed Germination at Cold and Warm Nonoptimal Temperatures

**DOI:** 10.1111/pce.70103

**Published:** 2025-08-05

**Authors:** Tina Steinbrecher, Antje Voegele, Michael Ignatz, Karin Weitbrecht, Safina Khan, Kai Graeber, James E. Hourston, Gerhard Leubner‐Metzger

**Affiliations:** ^1^ Department of Biological Sciences, Seed Biology and Technology Group Royal Holloway University of London Egham UK; ^2^ Institute for Biology II, Botany/Plant Physiology University of Freiburg, Faculty of Biology Freiburg Germany; ^3^ Eden Research plc Milton Park Oxfordshire UK; ^4^ Laboratory of Growth Regulators, Faculty of Science Palacký University and Institute of Experimental Botany, Czech Academy of Sciences Olomouc Czech Republic

**Keywords:** cell‐wall remodelling genes, endosperm weakening, molecular biomechanics, seed germination temperature, seed–environmental interaction, thermal‐time modelling, tissue elasticity/stiffness

## Abstract

Temperature sensing to adjust developmental rates and phenological responses to different climatic environments is critical for plant survival. Population‐based thermal‐time threshold models predict linear relationships between temperature and, for example, seed germination rates (speed), but the mechanisms are not known. Here, we used an integrative approach into the molecular biomechanical mechanisms underpinning a thermal‐time model by combining *Lepidium sativum* micropylar endosperm (CAP) and radicle transcriptome analysis at defined heat units (generated by different time‐temperature combinations) with corresponding CAP biomechanics. The thermal‐time model delivered linear relationships with germination rates, but the underpinning biomechanical mechanisms of CAP weakening differed fundamentally between the optimal (24°C–27°C), sub‐optimal (colder: 11°C, 18°C) and supra‐optimal (warmer: 32°C) temperatures. Chilling (11°C) differed from other temperatures in that its CAP weakening inhibition was combined with altered CAP stiffness/elasticity. Differentially expressed cell wall remodelling protein (CWRP) genes associated with CAP weakening and/or stiffness/elasticity were identified using defined heat unit comparisons. Xyloglucans, galactomannans, pectins and UDP‐sugar metabolism were major targets. Temperature regulation of CAP CWRP expression by DELAY‐OF‐GERMINATION‐1 (DOG1) controls CAP weakening and seed germination. We conclude that distinct and temperature‐specific molecular and biomechanical mechanisms underpin the apparently linear thermal‐time responses during CAP weakening and seed germination.

## Summary Statement

Plant seeds use molecular and mechanical processes to respond to cold and warm temperatures during germination. Their endosperm tissue is sensing the surrounding temperature and controls if and how quickly seeds germinate by weakening its cell walls in a temperature‐specific way. Seeds are ideal to study how organisms respond to temperature changes, offering insights into how species adapt to changing climates.

## 1 Introduction

Thermal‐time (‘temperature‐sum’, ‘heat‐sum’, ‘degree‐day’) models are widely used within agriculture, forestry and applied ecology to predict the seasonal timing of biological events, such as seed germination, egg hatching, flowering of plants, embryogenesis of nematodes, budburst in trees, appearance of pest insects, weed emergence and crop harvest dates (Batlla and Benech‐Arnold 2015; Johansson and Bolmgren 2019; Trudgill et al. 2005; Zhang et al. 2024b). Predicting phenological responses, the timing of life cycle events in different seasons and climatic environments, is also important for understanding how organisms, populations and species will respond to future climate change (Donohue et al. 2015; Fernández‐Pascual et al. 2019). Within populations, individuals often vary widely in their developmental responses to environmental factors. Population‐based threshold models specifically incorporate variation among individuals to predict these responses to the environment. Beyond predicting phenology, population‐based threshold models therefore have the potential to provide insight into ecological and evolutionary processes, but this requires unravelling the unknown underpinning molecular mechanisms. Seed biology is based on quantitatively analysing populations of individual seeds. Ambient temperature is the key environmental factor which controls seed germination timing in moist soil (Batlla and Benech‐Arnold 2015; Fernández‐Pascual et al. 2019; Finch‐Savage and Footitt 2017; Graeber et al. 2014; Hernando et al. 2021). Seeds therefore provide exellent systems for studying the mechanisms which are underpinning thermal‐time models.

Population‐based threshold thermal‐time models for germination describe the time courses of seed populations at different constant temperatures based on the concept that the base temperature (*T*_b_, minimum threshold below which germination will not occur) is usually constant among individual seeds of the population (Alvarado and Bradford 2002; Bierhuizen and Wagenvoort 1974; Bradford and Bello 2022; Ellis et al. 1986; Garcia‐Huidobro et al. 1982; Hegarty 1973; Loades et al. 2023; Rowse and Finch‐Savage 2003; Trudgill et al. 2005; Watt and Bloomberg 2012). Above *T*_b_, the rate (speed) of germination increases up to a temperature optimum (*T*_o_), above which it decreases and eventually ceases at the ceiling (maximal) temperature (*T*_c_). In a typical thermal‐time model linear relationships are obtained between imbibition temperature and germination rate (GR_g%_), that is, the inverse of the time to complete germination for a given percentage of the seed population (1/*t*_g%_). The estimated cardinal temperatures (*T*_b_, *T*_o_, *T*_c_; in °C) and thermal‐time constants (*θ*_cold_ and *θ*_warm_; in °C·h) are sufficient to predict germination timing for all percentage fractions of the seed population. In the sub‐optimal (cold, < *T*_o_) temperature range between *T*_b_ and *T*_o_, the lines representing different fractions vary in slope (*θ*_cold(g%)_ is normally distributed around *θ*_cold(50%)_) and have a common intercept at *T*_b_. In the supra‐optimal (warm, > *T*_o_) temperature range between *T*_o_ and *T*_c_, the lines are parallel (*θ*_warm_ is constant) and *T*_c_ is normally distributed around *T*_c(50%)_. An above threshold linear relationship between temperature and GR_g%_ for seed germination, or rate of development for other processes, is fundamental to thermal‐time models.

Seed germination starts with water uptake by imbibition of the dry seed and ends when the radicle has protruded all seed covering layers (Nonogaki 2019; Steinbrecher and Leubner‐Metzger 2017; Weitbrecht et al. 2011). These covering layers are typically the dead testa (seed coat) and a living endosperm of variable abundance which most angiosperms have retained in their mature seeds. Seed germination of the Brassicaceae relatives *Lepidium sativum* (garden cress) and *Arabidopsis thaliana* consists of two sequential steps: testa rupture which is subsequently followed by endosperm rupture, the visible completion of germination. Weakening of the micropylar endosperm covering the radicle and localized expansion growth of cells in the embryonic axis (growth zone: radicle plus lower hypocotyl) are concurrent processes preceding endosperm rupture (Bethke et al. 2007; Bewley 1997; Finch‐Savage and Leubner‐Metzger 2006; Nonogaki 2019; Steinbrecher and Leubner‐Metzger 2017; Toorop et al. 2000; Yan et al. 2014b). Endosperm weakening is required for the completion of germination and is associated with the gibberellin‐induced expression of cell wall remodelling proteins (CWRPs) in the micropylar endosperm (Bewley 1997; Chen et al. 2002; Dahal et al. 1997; Feurtado et al. 2001; Morris et al. 2011; Scheler et al. 2015; Steinbrecher and Leubner‐Metzger 2017; Voegele et al. 2011; Zhang et al. 2024b). *L. sativum* has emerged as the Brassicaceae model system for studying the molecular biomechanics of endosperm weakening as the large seed size allows the direct quantification of the tissue resistance by the puncture‐force method (Graeber et al. 2014; Lee et al. 2012; Linkies et al. 2009; Müller et al. 2009; Müller et al. 2006). These studies demonstrated that *L. sativum* micropylar endosperm weakening is strictly associated with germination timing. Environmental factors and hormones regulate the expression of downstream CWRP‐encoding genes which alter the mechanical strength of the micropylar endosperm.

Plant cell walls are ridged extra‐protoplasmatic structures and provide, together with the turgor pressure from water uptake into the cell vacuole, the mechanical stability to cells, tissues or organs. The primary cell wall is a biological composite material consisting mainly of cellulose, hemicelluloses (e.g., xyloglucan, xylan), pectins (e.g., xylogalacturonan, homogalacturonan, rhamnogalacturonan) and proteins (Burgert and Keplinger 2013; Franková and Fry 2013; Gigli‐Bisceglia et al. 2020; Pauly and Keegstra 2016). Cell wall and cell expansion growth, as well as intercellular adhesion and separation of cell walls in tissues, is a fundamental feature of plant growth and development such as endosperm weakening during seed germination (Bethke et al. 2007; Cosgrove 2016, 2018; Daher and Braybrook 2015; Levesque‐Tremblay et al. 2015; Yan et al. 2014b). Seeds provide excellent study systems for investigating how plant cells, tissues and organs growth depend on altered mechanical properties of their cell walls in response to environmental cues (Le Gall et al. 2015; Steinbrecher and Leubner‐Metzger 2017; Tenhaken 2015) including non‐optimal temperatures. The *DELAY OF GERMINATION1 (DOG1)* gene defines the temperature window permitting seed germination by affecting the expression of CWRP genes required for the temperature‐dependent control of micropylar endosperm weakening (Graeber et al. 2014).

The endosperm of imbibed seeds is involved in temperature sensing to mediate temperature‐dependent germination responses via transcriptome changes (Graeber et al. 2014; Hernando et al. 2021; Yan et al. 2014b). For the mechanisms underpinning thermal‐time models, a linear regulation of enzymatic activities by temperature has been proposed rather than a single, rate‐limiting enzyme which weakens micropylar endosperm cell walls (Bewley 1997; Dahal et al. 1997; Finch‐Savage and Leubner‐Metzger 2006; Hegarty 1973; Trudgill et al. 2005). Whether transcriptome changes with thermal‐time regulation of CWRP gene expression generate the observed linear relationship between temperature and seed germination rates has so far not been investigated. Here, we use the non‐dormant seeds of *L. sativum* as an excellent system to investigate the biomechanical and molecular mechanisms by which ambient temperature controls micropylar endosperm weakening. We integrate thermal‐time modelling with biomechanical and transcriptome analysis to investigate if the observed linear relationship between temperature and germination speed is caused by temperature‐mediated gradual accleration and deceleration of the same processes or if distinct temperature‐specific mechanisms underpin the apparently linear thermal‐time responses during endosperm weakening and seed germination.

## 2 Materials and Methods

### 2.1 Plant Material and Germination Assays

An after‐ripened seed batch of *Lepidium sativum* L. cultivar FR14 (‘Keimsprossen’, Juliwa) was used as in earlier work (Graeber et al. 2011; Scheler et al. 2015). *L. sativum* FR14 seeds over‐expressing the *DELAY OF GERMINATION 1 (DOG1)* gene under the control of the CaMV 35S‐promoter, transgenic lines Lesa‐OxAtDOG1‐E17 and ‐B13, were used in this study. They were characterized by DOG1 protein accumulation in seeds leading to a severely delayed germination phenotype, reduced germination speed and reduced optimal temperature from 24°C to 18°C when compared to wild type (Graeber et al. 2011). To analyze germination 50 seeds per replicate were placed into 9‐cm Petri dishes with two layers of filter paper with 6 mL 1/10 Murashige and Skoog (MS) inorganic salts and incubated in a MLR‐350 Versatile Environmental Test Chamber (Sanyo/Panasonic, Bracknell, UK) in continuous white light (~100 µmol m^−2^ s^−1^) at the temperatures indicated; corresponding 4°C plates were incubated on ice. For the thermal‐time modelling, 25 seeds per replicate were placed into 5.3‐cm Petri dishes with one layer of filter paper with 3 mL 1/10 MS inorganic salts and incubated on a temperature table producing a gradient of temperatures by heating on one side and cooling on the opposed side (continuous white light ~20 µmol m^−2^ s^−1^). Testa (TR) and endosperm rupture (ER) were scored for three‐five replicate plates using a binocular microscope (Scheler et al. 2015). Three biological replicates, that is, plates each with 50 (Versatile Environmental Test Chamber) or 25 (temperature table) seeds, were used in the germination experiments.

### 2.2 Population‐Based Thermal‐Time Threshold Modelling

The cardinal temperatures permissible for germination including base temperature (*T*_b_), optimal temperature (*T*_o_) and ceiling (maximal) temperature (*T*_c_), and the thermal‐time constants Θ were identified by population‐based threshold modelling for thermal‐time (Alvarado and Bradford 2002; Batlla and Benech‐Arnold 2015; Bierhuizen and Wagenvoort 1974; Bradford and Bello 2022; Finch‐Savage and Leubner‐Metzger 2006; Garcia‐Huidobro et al. 1982) as described in detail by Loades et al. (2023). In brief, germination rates GR_g_%, that is, the inverse of time to germination (ER) for a given percentage of the population (1/*t*_g_%), were plotted against the imbibition temperatures for the 10%–90% fractions. Linear relationships between the germination rates (GR_g%_) were obtained within the sub‐optimal (between *T*_o_ and *T*_b_) and supra‐optimal (between *T*_o_ or *T*_c_) temperature ranges based on linear regression analysis to calculate the best goodness of fit (*R* ^2^) lines. *T*_b_ and *T*_c_ were estimated from their intercepts with the *x*‐axis. The estimated *T*_b_ fitting all fractions was used to recalculate regression lines forced through this *T*_b_ value for the linear lines in the sub‐optimal (colder) temperature range. The thermal‐time constants *θ*_cold(g)_ derived from the slopes of the different fractions were normal‐distributed around the mean (*θ*_cold(50%)_ ± SD). The estimated *T*_c(50%)_ and equal slopes were obtained for the linear lines in the supra‐optimal temperature range. The thermal time constant *Θ*_warm(g)_ derived from their slopes was constant. At supra‐optimal temperatures, *T*_c(g)_ is normal distributed around *T*_c(50%)_. All statistical analyses were performed in GraphPad Prism v10 (GraphPad Software Inc., San Diego, CA, USA).

### 2.3 Biomechanical Measurements

Puncture force measurements were conducted as described previously using a modified custom‐made biomechanics device (Graeber et al. 2014; Lee et al. 2012; Linkies et al. 2009; Pearce et al. 2018; Steinbrecher and Leubner‐Metzger 2017). In brief, intact micropylar endosperm (CAP) tissue was dissected from the imbibed seeds and glued to a metal sample holder (0.6 mm hole) using Loctite 454 glue (Henkel, Germany). CAP tissue was continuously incubated in 1/10 MS inorganic salts during dissection and drying (30 min) of the glue. All CAP tissues have the shape of half prolate spheroids and are equal in size and shape (Pearce et al. 2018). A rounded metal pin was driven into the sample while force and displacement were recorded simultaneously. A pin with 0.3 mm diameter was used at a speed of 0.7 mm min^−1^. The CAP puncture force (PF; tissue resistance or strength) and the tissue stiffness/elasticity were determined from the force‐displacement curves as the maximal forces and the slopes, respectively, as described in Figures 2B and S1. Puncture force and stiffness were measured at different time points and temperatures for seeds just before TR as well as for seeds after TR, as indicated.

### 2.4 RNA Isolation From Seed Tissues

Total RNA was extracted from 200 CAPs and 200 RADs (growth zone: radicle plus lower 1/3 hypocotyl) dissected from *L. sativum* seeds (Scheler et al. 2015) after 10 h of imbibition at different temperatures (11°C, 18°C, 27°C, 32°C) and frozen in liquid nitrogen. For collection of CAP and RAD tissues, only seeds without ER were used, and the ~50% percentage of TR present in the population was covered in the sampled tissue (Figure S1). RNA extraction was performed as described using our seed‐optimized procedure and followed by quantity and quality control (Graeber et al. 2011; Scheler et al. 2015). Four biological replicate RNA samples were used for all downstream applications.

### 2.5 RNA Microarray Hybridizations and Data Analysis

*L. sativum* RNAs were labelled and hybridized to GeneChip® Arabidopsis ATH1 Genome arrays from Affymetrix as described (Scheler et al. 2015). Microarray data are available in the ArrayExpress database (www.ebi.ac.uk/arrayexpress) under accession number E‐MTAB‐1811. Hybridization of *L. sativum* genomic DNA and determination of valid *A. thaliana* probes corresponding to *L. sativum* genes resulted in a total of 21,478 probes out of 24,000 probes present on the chip (89.5%) having significant hybridization. The masking was done using the Xspecies method as described by Scheler et al. (2015). Thresholds were calculated from ATH1 chips hybridized with *L. sativum* genomic DNA. Gene expression was further analyzed using the Analyst 7.5 Software (Genedata AG, Basel, Switzerland) to obtain lists of > 2‐fold differentially regulated genes (DEGs) as described (Scheler et al. 2015).

### 2.6 Quantitative (Real‐Time) RT‐PCR (RT‐qPCR) and Statistical Analyses

Normalized transcript abundances were analyzed by RT‐qPCR, using superior reference genes as described (Graeber et al. 2011; Scheler et al. 2015). First‐strand cDNAs were obtained using the Superscript III reverse transcriptase kit (Invitrogen) with 0.3 nmol random pentadecamers for 20 µL reverse transcription reactions of 5 µg of total RNA. The RT‐qPCR (for primers see Table S1) and post‐run data analysis and transcript abundances were normalized against the geometric mean of the three validated reference genes *LesaG17210* (HQ912755), *LesaG04660* (HQ912754) and *LesaG20000* (HQ912757) for each sample were conducted as described (Graeber et al. 2014; Graeber et al. 2011; Scheler et al. 2015). Statistical analyses of obtained germination, thermal‐time modelling, biomechanics and RT‐qPCR results were conducted using GraphPad Prism v10 (GraphPad Prism v10, Boston, MA, USA).

## 3 Results

### 3.1 Distinct Biomechanical Mechanisms Underpin Seed Temperature Responses

**Figure 1:**
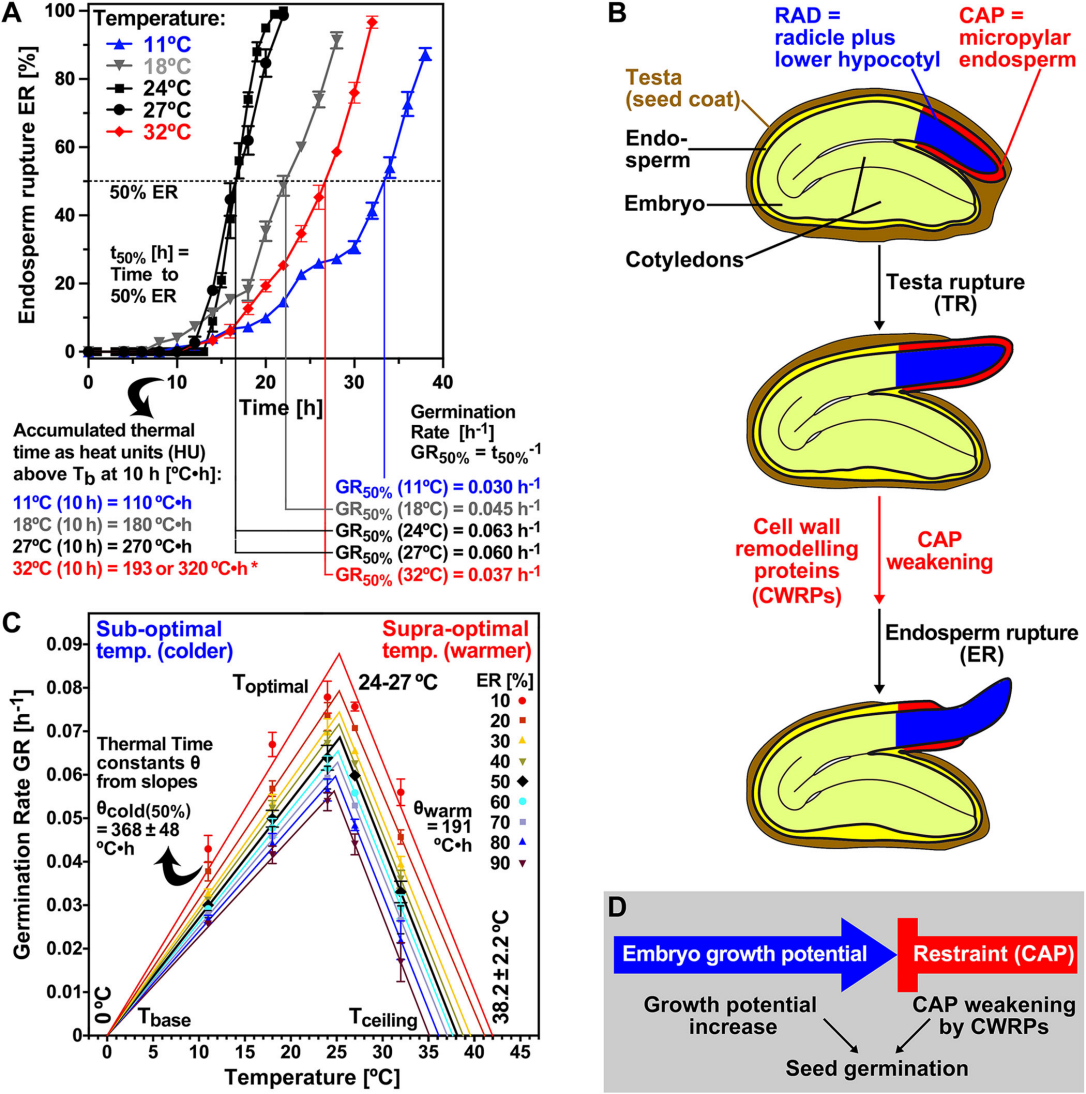
The effect of imbibition temperature on the germination of the non‐dormant *Lepidium sativum* FR14 seed population. (A) Time course of endosperm rupture (ER) of seeds incubated at 11°C, 18°C, 24°C, 27°C and 32°C in continuous white light. Mean values ± SE of 3 × 50 seeds. Calculation of Germination Rates at 50% ER (GR_50%_) are exemplarily shown. Accumulated heat units (HU) above *T*_b_ (0°C) are presented for 10 h, the sampling time for the transcriptome analysis; the * indicates the two alternative possibilities for accumulated thermal‐time at 32ºC (*see main text for details*). Note that in contrast to ER, the corresponding time course of testa rupture (TR) did not appreciably differ between the five temperatures until 50% TR (Figure S1). (B) Seed compartments (CAP, micropylar endosperm, *in red*; RAD, growth zone: radicle plus lower 1/3 hypocotyl, *in blue*) at different stages during germination as related to TR and ER; modified from Scheler et al. (2015) with permission. (C) Population‐based thermal‐time modelling of germination rates (GR_g%_ = 1/t_g%_) from the ER time courses (*panel A*). Optimal germination was in the 24°C–27°C region with T_o_ ~25.5°C. The base temperature *T*_b_ (0°C) was constant for all fractions, while the thermal‐time constants *θ*_cold_ were normal‐distributed (*θ*_cold(50%)_ ± SD). The ceiling temperature *T*_c_ was normal‐distributed (*T*_c(50%)_ ± SD) around the estimated 38.2°C value, while the thermal‐time constant *θ*_warm_ was constant for all fractions. (D) The completion of germination by ER and radicle emergence occurs when the increasing embryo growth potential (RAD) exceeds the restraint of the weakening micropylar endosperm (CAP).

Thermal responses of seed populations can be quantified in terms of ‘thermal‐time’ allowing predictions of germination timing at different imbibition temperatures (*see introduction*). Here we used population‐based threshold thermal‐time modelling of the non‐dormant seeds of *Lepidium sativum* FR14 (Graeber et al. 2014; Linkies et al. 2009; Müller et al. 2006; Scheler et al. 2015; Voegele et al. 2011) to estimate the cardinal temperatures (*T*_b_, *T*_o_, *T*_c_; in °C) and thermal‐time constants (*θ*_cold_ and *θ*_warm_, i.e., the inverse of the slopes of the regression lines; in °C·h). This provided a physiological framework for investigating the biomechanical and transcriptomic mechanisms underpinning the responses to sub‐optimal (colder), optimal and supra‐optimal (warmer) temperatures (Figure 1). The two sequential visible events during germination, testa rupture (TR) and endosperm rupture (ER), were scored over time at 11°C, 18°C, 24°C, 27°C and 32°C (Figures 1A,B, S1). Figure 1A shows that both, the colder (11°C, 18°C) and the warmer (32°C) temperatures, delayed the times required for the seed populations to reach 50% ER (*t*_50%_) compared to the optimal 24°–27°C temperature range. Germination rates (GRs), providing quantitative ‘speed values’ characterizing the seed population were calculated, are exemplarily depicted for GR_50%_ in Figure 1A.

For the thermal‐time modelling, the GRs were plotted against the imbibition temperatures (Figure 1C). Optimal germination for the *L. sativum* seed population was in the 24°C–27°C region with *T*_o_ ~25.5°C. Linear relationships were evident for each fraction within the suboptimal (colder, between *T*_b_ and *T*_o_) and supra‐optimal (warmer, between *T*_o_ and *T*_c_) temperature ranges (Figure 1C). The base temperature *T*_b_ (0°C) for FR14 was constant for all fractions, very similar to the published value for another *L. sativum* cultivar (1°C, Bierhuizen and Wagenvoort 1974). *θ*_cold_ derived from the slopes of the regression lines was normal‐distributed around *θ*_cold(50%)_ (Figure 1C). In the supra‐optimal temperature range the regression lines were parallel (*θ*_warm_ was constant) and *T*_c_ was normal‐distributed around *T*_c(50%)_ (38.2°C). The accumulated thermal‐time above *T*_b_ at 10 h was therefore 110°C·h (at 18°C), and 270°C·h (at 27°C) in the suboptimal to optimal temperature range. The decrease in GR values in the supra‐optimal temperature range is known to be associated with temperature‐dependent changes in seed water relations (Bradford and Bello 2022), leading to alternative possibilities to accommodate this in the model: (1) Rowse and Finch‐Savage (2003) proposed that thermal‐time continues to accumulate above To and is offset by the concurrent increase in base water potential; (2) Alvarado and Bradford (2002) proposed that the accumulation of thermal‐time ceases at *T*_o_ and effectively decreases between *T*_o_ and *T*_c_. The accumulated thermal‐time above *T*_b_ at 10 h at 32°C (Figure 1) could therefore either be calculated as 320°C·h (possibility 1) or as 193°C·h (possibility 2). We used this physiological framework (Figure 1) with similarly reduced GR values in the nonoptimal, colder (11°C, 18°C) or warmer (32°C) ranges compared to *T*_o_, to investigate if the underpinning molecular and biomechanical mechanisms of endosperm weakening and rupture are simply driven by thermal‐time (accumulation of heat units above *T*_b_ in °C·h) or by temperature‐specific processes.

**Figure 2:**
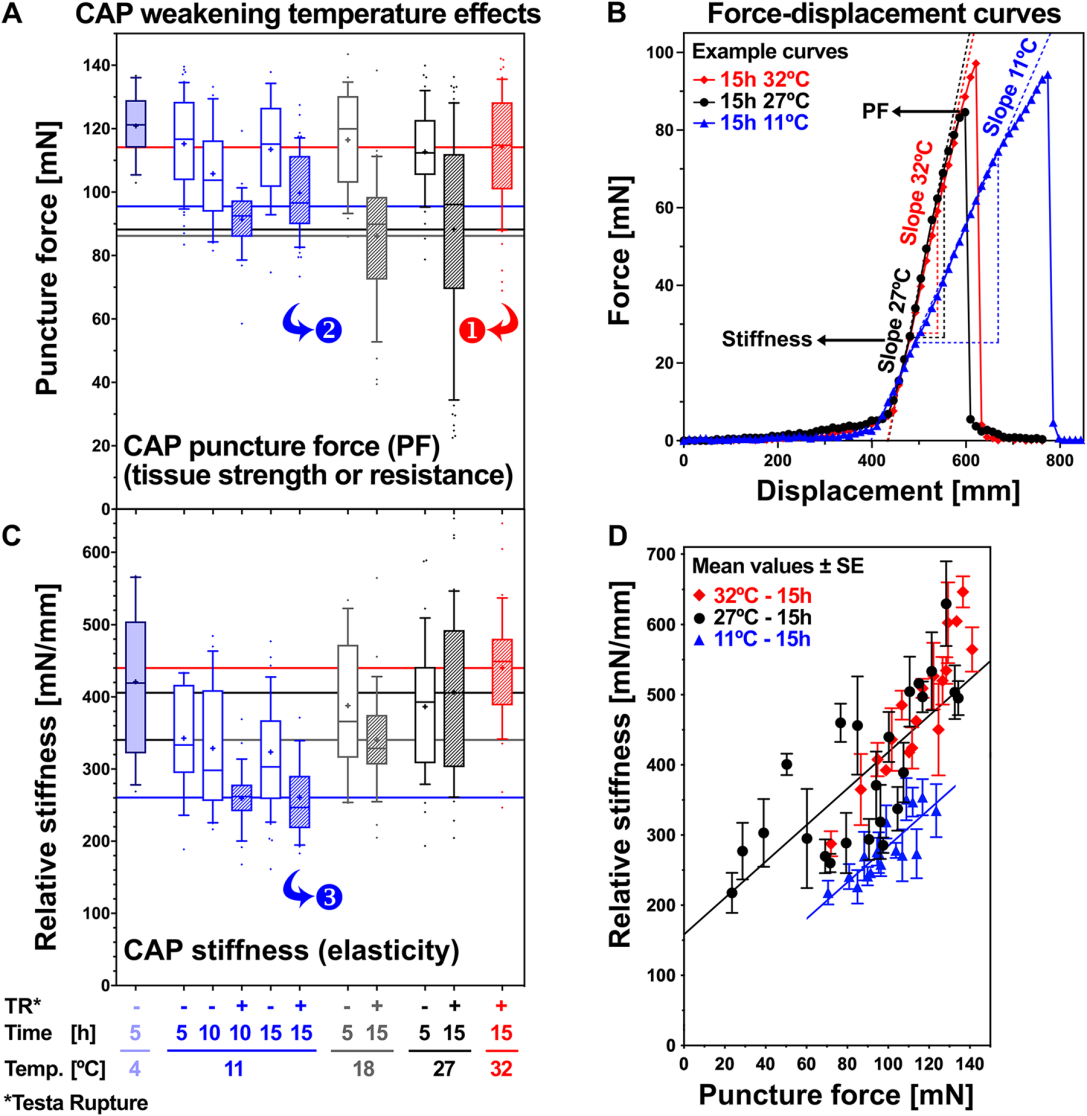
The effect of imbibition temperature on the dynamic biomechanical properties of *Lepidium sativum* micropylar endosperm (CAP) weakening. (A) Puncture‐force (PF tissue strength or resistance) measurement of CAPs dissected at the times and temperatures indicated during seed imbibition, from seeds without (−TR) and with (+TR) testa rupture. Data of 20–60 CAPs are shown as box‐whisker plots, representing the minimum and the maximum of the data distribution (top: 25th percentile, bottom: 75th percentile, horizontal bar: median, cross: mean). Lines indicate the PF mean values at 15 h for 11°C ± TR (*blue*), 18°C (*grey*), 27°C (*black*), 32°C (*red*). ANOVA *p* values for temperature comparisons at 15 h: 18°C–11°C (−TR) 0.0003, 18°C–11°C ( + TR) n.s., 27°C–11°C (−TR) 0.0003, 27°C–11°C (+TR) n.s., 32°C–11°C (−TR) n.s., 32°C–11°C (+TR) 0.013, 27°C–18°C n.s., 32°C–18°C < 0.0001, 32°C–27°C < 0.0001. Arrows and numbers indicate the type of identified CAP cell wall remodelling protein (CWRP) differentially expressed genes (DEG) in the transcriptome analysis (*see main text for details*). (B) Representative force–displacement curves for single puncture force measurements of CAPs dissected at 15 h at the temperatures indicated. PF values (*panel A*) were derived from the maximum forces of these curves. (C) Relative CAP stiffness (elasticity) values displayed in as Box–Whisker plots, values are derived from the slopes of the linear (elastic) region of the force–displacement curves (*see panel B*) of > 20 measurements of CAPs dissected at the times and temperatures indicated, from seeds −TR or +TR, as indicated. Lines indicate the stiffness mean values at 15 h for 11°C ± TR (*blue*), 18°C (*grey*), 27°C (*black*), 32°C (*red*). ANOVA *p* values for temperature comparisons at 15 h: 18°C–11°C (−TR) n.s., 18°C–11°C (+TR) < 0.05, 27°C–11°C (−TR) 0.0006, 27°C–11°C (+TR) < 0.0001, 32°C–11°C (–TR) < 0.0001, 32°C–11°C (+TR) < 0.0001, 27°C–18°C < 0.05, 32°C–18°C 0.0007, 32°C–27°C n.s. (D) Mean ± SE values provided linear relationships at 15 h (+TR) between decreasing CAP stiffness and decreasing CAP puncture force at all temperatures. Mean ± SE values presented were each obtained from 3 consecutive data points (single values) from lists of all data points for each series, sorted in ascending order. Note that the regression line for 11°C exhibited a parallel translation downwards by ~150 mN/mm compared to the 27°C regression line. In contrast to this, the 32°C and 27°C 15 h values were not statistically different, and the 5 h stiffness values (unweakened) CAPs were not statistically different at any temperature. ANOVA *p* values for temperature comparisons at 15 h (force range 75–125 mN): 18°C–11°C < 0.01, 27°C–11°C < 0.001, 32°C–11°C < 0.001, 27°C–18°C < 0.05, 32°C–18°C < 0.001, 32–27 n.s.

Biomechanical analysis of *L. sativum* micropylar endosperm (CAP *hereafter*) weakening demonstrated that there was a correlation between the decreasing CAP puncture force (mechanical strength of the CAP tissue) and ER (Figures 1B,D and Linkies et al. 2009; Müller et al. 2006; Steinbrecher and Leubner‐Metzger 2017). Unweakened CAPs at 5 h had puncture force values ~115 mN, and for 18°C and 27°C CAP weakening was evident at 15 h with puncture force values decreased to 80–85 mN (Figure 2A); this was also evident at 24°C (Graeber et al. 2014). The CAP puncture force (PF) is the maximum force value derived from the force‐displacement curves (Figure 2B) and provides a quantitative measure of the CAP tissue strength. In contrast to the optimal temperatures (24°C, 27°C) and 18°C, CAP weakening was inhibited at supra‐optimal and sub‐optimal temperatures with no (32°C) or smaller (11°C) reduction in puncture force values, respectively (Figure 2A). The results at 11°C were of particular interest as a comparably small reduction in the puncture force, to ~95 mN, was strictly associated with TR. In contrast to this, at 32°C CAPs remained unweakened although they completed TR.

Biomechanical tests with plant cell walls demonstrated that depending on their biochemical composition they differ in strength and/or in stiffness (elasticity) as key mechanical properties (Burgert and Keplinger 2013; Cosgrove 2018; Steinbrecher and Leubner‐Metzger 2017), but stiffness was so far not analysed in germinating seeds. CAP stiffness derived from the slope of the force‐displacement curves (Figure 2B) and provides a quantitative measure for the CAP tissue elasticity. Interestingly, while they differed significantly in their puncture force values (Figure 2A), weakened 27°C and unweakened 32°C 15‐h CAPs did not differ in their stiffness (Figure 2C). In contrast to this, 11°C CAPs became more elastic over time, with reduced stiffness evident at the 10‐ and 15‐h time points. The reduced stiffness was already evident before TR and was further reduced upon TR (Figures 2C and S1). Figure 2D shows that there was a linear relationship at 15 h between decreasing CAP stiffness and decreasing puncture force at all temperatures, but the regression line for 11°C exhibited a parallel translation downwards by ~150 mN/mm compared to the 27°C regression line, while the 32°C and 27°C stiffness values were not statistically different (Figure 2D). We conclude from these findings that puncture force and stiffness changes are both biomechanical hallmarks of endosperm CAP weakening; and further, that the underpinning biomechanical mechanisms differ fundamentally between the optimal, supra‐optimal (warmer) and sub‐optimal (colder) temperatures, as well as within the sub‐optimal temperature range.

### 3.2 Comparative Thermal‐Time Analysis of Transcriptomes During Endosperm CAP Weakening

The finding that the completion of germination by ER of our *L. sativum* seed population at different imbibition temperatures provided a typical thermal‐time model with a common *T*_b_ (0°C, Figure 1), but distinct biomechanical mechanisms (Figure 2), raised the intriguing question to which extent is the underpinning gene expression thermal–time compliant. We demonstrated earlier that biochemical cell wall, hormonal and transcript abundance changes in the CAP (micropylar endosperm) and RAD (radicle plus lower 1/3 hypocotyl) compartments (Figure 1B) are associated with CAP weakening, RAD expansion and ER, and further that the balance between CAP restraint and RAD growth potential (Figure 1D) determines germination timing (Graeber et al. 2014; Linkies et al. 2009; Morris et al. 2011; Müller et al. 2009; Scheler et al. 2015; Steinbrecher and Leubner‐Metzger 2017; Voegele et al. 2011). To investigate to which extent transcript abundances are expressed in compliance with thermal‐time, we compared the CAP and RAD transcriptomes of seeds imbibed for different times (5, 7, 10, 13 h) at 24°C (‘time course microarrays’, Scheler et al. 2015) with the CAP and RAD transcriptomes of seeds imbibed at different temperatures (11°C, 18°C, 27°C, 32°C) at 10 h (‘temperature microarrays’). *L. sativum* transcripts are referred to by the microarray probes of the putative *Arabidopsis thaliana* orthologs which have an AGI identifier and annotation linked to it (www.Arabidopsis.org). In the ‘temperature arrays’ normalized expression data were obtained for 20,775 *L. sativum* transcripts (SI Dataset S1).

**Figure 3:**
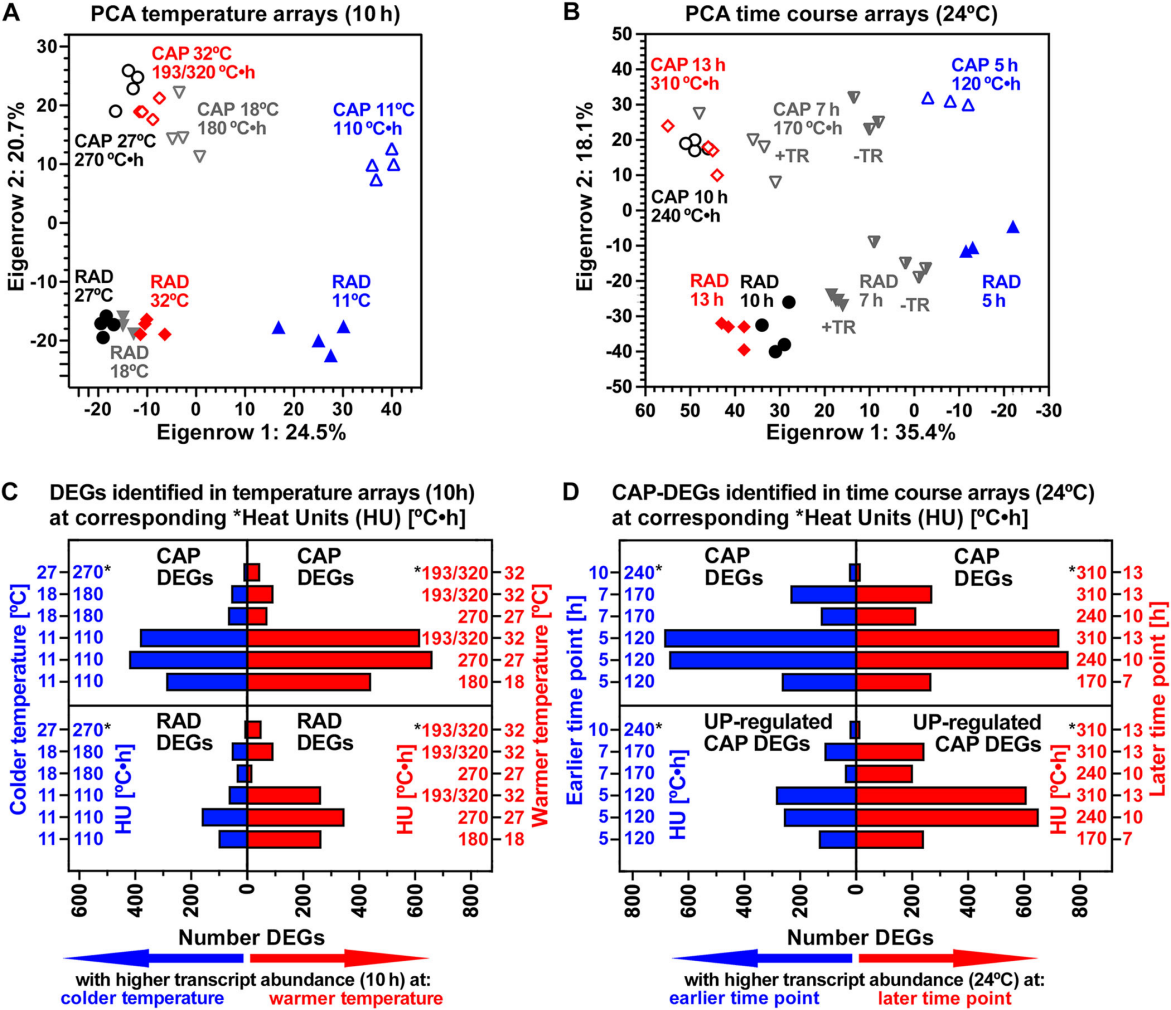
Temperature and time course transcriptome analysis of *Lepidium sativum* micropylar endosperm (CAP) weakening and radicle (RAD) expansion growth. (A) Principal component analysis (PCA) of the ‘temperature arrays’ comparing the transcriptomes of the CAP (micropylar endosperm) and RAD (radicle plus lower 1/3 hypocotyl, embryo growth zone) compartments at 10 h at 11°C, 18°C, 27°C and 32°C. Heat unit (HU, in °C·h) values (above *T*_b_ = 0°C) are indicated for the CAP time‐temperature combinations; note that two alternative possibilities for calculating accumulated thermal‐time at 32°C lead to either 193°C·h or 320°C·h (*see main text for details*). (B) PCA of the ‘time course arrays’ comparing the transcriptomes of the CAP and RAD compartments at 24°C over time (5, 7, 10 and 13 h); see Scheler et al. (2015) for details. HU values are indicated for the CAP time‐temperature combinations. (C) Comparison of numbers of differentially expressed genes (DEGs) in the CAP and RAD temperature transcriptomes at the HU values calculated from the four temperatures at 10 h. In total, 1468 CAP and 773 RAD DEGs (≥ 2‐fold cut‐off) were identified in the various HU comparisons with higher transcript abundances at either colder (620 CAP DEGs) or warmer (918 CAP DEGs) temperatures, corresponding to lower and higher HU values, respectively. The identified DEGs and their transcript abundances are listed in SI Datasets S2 (110 vs. 180°C·h), S3 (110 vs. 270°C·h), S4 (110 vs. 193/320°C·h), S5 (180 vs. 270°C·h), S6 (180 vs. 193/320°C·h) and S7 (270 vs. 193/320°C·h). (D) Comparison of numbers of CAP DEGs (≥ 2‐fold cut‐off) in the time course transcriptomes at the HU values calculated from the times (5, 7, 10 and 13 h) at 24°C. In total, 2697 CAP DEGs were identified in the various HU comparisons with higher transcript abundances at either earlier or later time points (*top panel*, SI Dataset S8). Of these, 1499 DEGs were identified as Upregulated CAP DEGs in the time course transcriptomes when compared to the dry seed state (0–1 h time points) values (*bottom panel*, SI Dataset S9). Higher transcript abundances were observed for these UP‐regulated CAP DEGs either at earlier (541 DEGs) or later (1003 DEGs) time points, corresponding to lower or higher HU values, respectively.

Principal component analysis (PCA) was used to reduce dimensionality of the data to identify global patterns for the comparisons across all transcripts (Figure 3). In the temperature transcriptomes, the eigenvectors with the largest influence represent imbibition temperature and seed compartment (Figure 3A). Eigenrow 2 (20.7%) clearly separated CAP and RAD, which is consistent with the *L. sativum* seed time course transcriptomes at 24°C (Figure 3B and Scheler et al. 2015). In the temperature transcriptomes the variance in gene expression differed considerably between the two key compartments at any temperature (Figure 3A). Eigenrow 1 (24.5%) separated the temperatures: 27°C, 32°C and 18°C cluster together, while 11°C cluster further away. The largest distance was observed between the 27°C and 11°C transcriptomes for both compartments, demonstrating that the greatest thermal transcript expression changes occurred at 11°C compared to the optimal temperature (27°C). Using accumulated heat units above T_b_ (HU), the 11°C, 18°C, 27°C and 32°C temperatures at 10 h correspond to 110, 180, 270 and 193/320°C·h, respectively (Figure 3A,C). For the germination transcriptome time course at 24°C, the 5, 7, 10 and 13 h time points delivered 120, 170, 240, and 310°C·h as HU values, respectively (Figure 3B,D). The PCAs of the time course and the temperature transcriptomes had a similar pattern for CAP and RAD when compared on a HU basis (Figure 3A,B). As for the temperature transcriptome 270°C·h (*T*_o_) versus 110°C·h (11°C) comparison (Figure 3A), the greatest transcript expression differences occurred in the time course transcriptomes for the 240°C·h versus 120°C·h comparison (Figure 3B).

Transcript abundance differences in the time course and temperature transcriptomes were used to identify gene candidates differentially expressed at various contrasting HU value comparisons (Figure 3). To identify DEGs in the temperature transcriptomes statistical significance of mean transcript abundance differences between HU values (representing different temperatures at 10 h) and a cut‐off of ≥ 2‐fold were set as criteria for differential regulation (SI Datasets S2–, S7). In total, 1468 CAP and 773 RAD DEGs were identified in the various HU value comparisons (Figure 3C). As expected from the PCA results, the largest number of DEGs was observed for 11°C (110°C·h) versus 27°C (270°C·h). The 32°C (193/320°C·h) versus 27°C (270°C·h) comparison resulted in the smallest number of CAP DEGs (Figure 3C). By using the same criteria as for the temperature transcriptomes, normalized expression data for 13,895 *L. sativum* CAP transcripts of the time course transcriptomes at 24°C (Scheler et al. 2015) delivered in total 2697 CAP DEGs (Figure 3D, *top panel*; SI Dataset S8). Comparison of their transcript abundances in imbibed seeds with the dry seed state identified among these are 1499 upregulated CAP DEGs (Figure 3D, *bottom panel*; SI Dataset S9).

**Figure 4:**
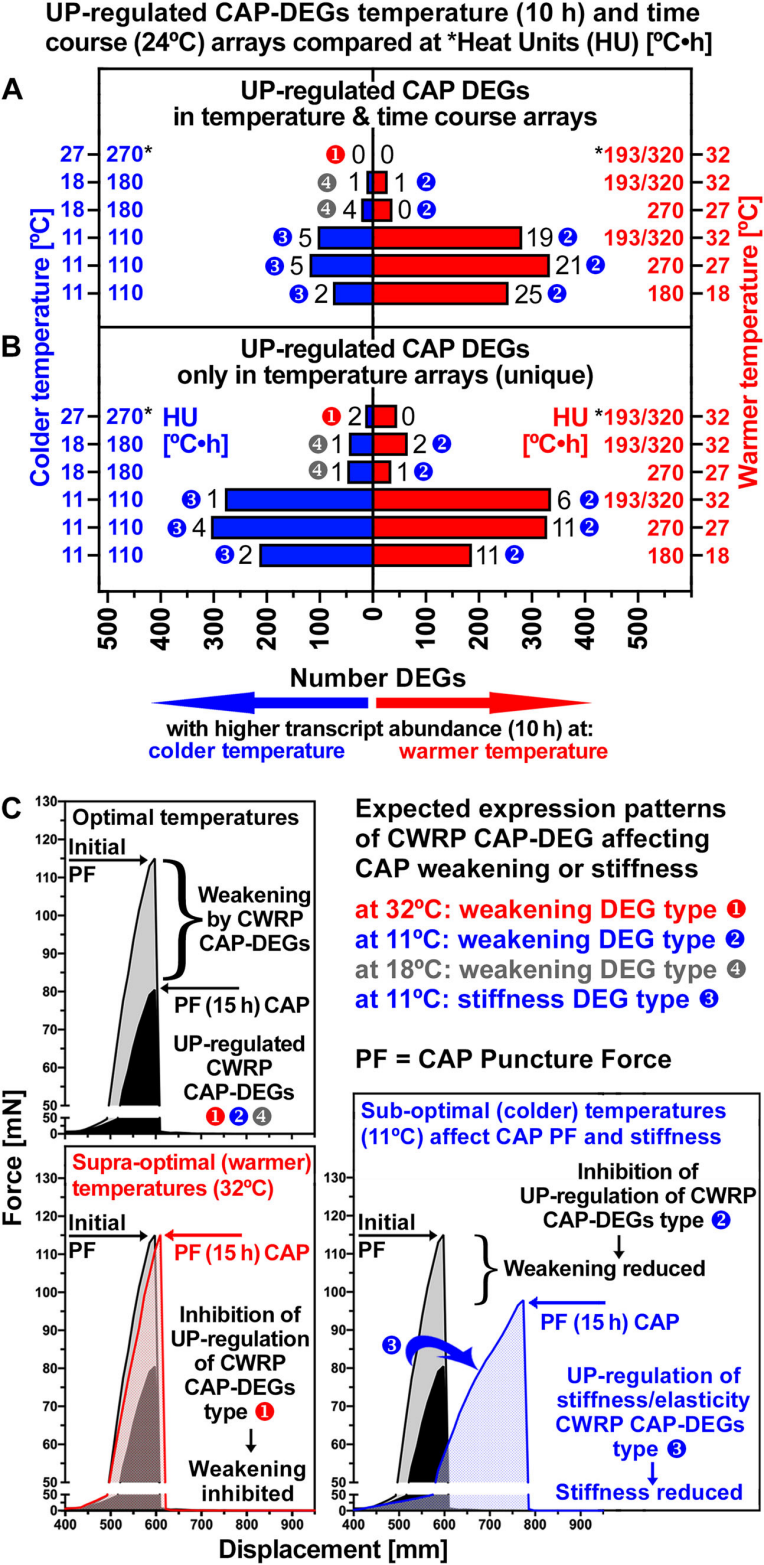
Identification of *Lepidium sativum* CAP weakening and stiffening cell wall remodelling protein (CWRP) DEGs in the temperature and time course transcriptomes. (A) Comparison of the numbers of UP‐regulated CAP DEGs at the various HU values which are shared between the temperature and time course transcriptomes. A total of 553 CAP DEGs common to both transcriptomes were identified (SI Dataset S10). Of these 37 were shared CAP CWRP DEGs (*numbers next to the bars*; SI Dataset 13). (B) Comparison of the numbers of upregulated CAP DEGs at the various HU values in the temperature transcriptomes only, that is, these were no DEGs in the time course transcriptomes. A total of 991 CAP DEGs unique to the temperature transcriptomes were identified (SI Dataset S11). Of these 24 were unique CAP CWRP DEGs (*numbers next to the bars*; SI Dataset S13). In addition, 58 UP‐regulated CWRP CAP DEGs were unique to the time course transcriptomes (Figure S2). (C) Biomechanics of CAP weakening at optimal temperatures reduced the initial (5 h) puncture force (PF, tissue strength, maximum force in the depicted force–displacement curves) to a lower value (15 h) by the action of UP‐regulated CAP CWRP DEGs. Different types of CAP weakening DEGs were distinguished based on their distinct expression patterns in the temperature microarrays at 10 h compared to 27°C (transcript abundance ratios ≥ 2 or ≤ 0.5): type‐1 (down at 32°C), type‐2 (down at 11°C), type‐3 (up at 11°C), type‐4 (up at 18°C). Expected expression patterns of these CAP CWRP DEGs for affecting CAP weakening or stiffness.

Cross‐comparison of these DEG lists revealed that the temperature and time course transcriptomes shared 155 upregulated CAP DEGs at lower HU values (colder temperatures in temperature arrays corresponding to earlier time points in time course arrays) and 398 upregulated CAP DEGs at higher HU values (warmer temperatures in temperature transcriptomes corresponding to later time points in time course transcriptomes), a total of 553 DEGs (Figure 4A, *top panel*; SI Dataset S10). A total of 991 upregulated CAP DEGs were unique to the temperature transcriptomes, with the majority associated with the 11°C temperature (Figure 4B, *bottom panel*; SI Dataset S11). These were 471 upregulated CAP DEGs at lower HU values (colder temperatures) and 520 upregulated CAP DEGs at higher HU values (warmer temperatures). The comparison of these DEG lists enabled to identify putative cell wall remodelling protein (CWRP) genes during CAP weakening at the various HU value comparisons which were either shared DEGs between the temperature and time course transcriptomes (37 in total, *numbers next to the bars in* Figure 4A, SI Dataset S12), specific only to the temperature transcriptomes (24 in total, *numbers next to the bars in* Figure 4B, SI Dataset S12), or specific only to the time course transcriptomes (58 in total, Figure S2).

Different types of CWRP CAP DEGs were distinguished based on their expression patterns in the temperature transcriptomes: In the optimal temperature range the reduction in puncture force during CAP weakening (Figure 2) was associated with the upregulation of type‐1, type‐2 and type‐4 DEGs (Figure 4C). Type‐1 CWRP CAP‐DEG expression was inhibited at 32°C which inhibited the decrease in CAP puncture force at the supra‐optimal temperature. Type‐2 CWRP CAP‐DEG expression was inhibited at 11°C which inhibited the decrease in CAP puncture force in the sub‐optimal chilling temperature. Compared to the optimal temperature, type‐3 and type‐4 CWRP CAP‐DEGs were upregulated by 11°C and 18°C, respectively. The expression pattern of type‐3 CWRP CAP‐DEGs at 11°C provided stiffness/elasticity gene candidates (Figure 4C). These expression patterns of identified CWRP CAP‐DEGs based on HU value comparison in the temperature and time course transcriptomes (Figure 4, SI Datasets S12 and 13) were analysed below with regard to the question if their expression is regulated by thermal‐time. Thermal‐time compliant DEG regulation patterns will provide equal transcript abundances at equal HU values generated by different time‐temperature combinations, while unequal transcript abundances will indicate temperature‐specific DEG regulation.

### 3.3 Thermal‐Time and DOG1 Regulation of Expansins and CAP Weakening Genes Encoding Galactomannan and Cellulose Targeting Enzymes

**Figure 5:**
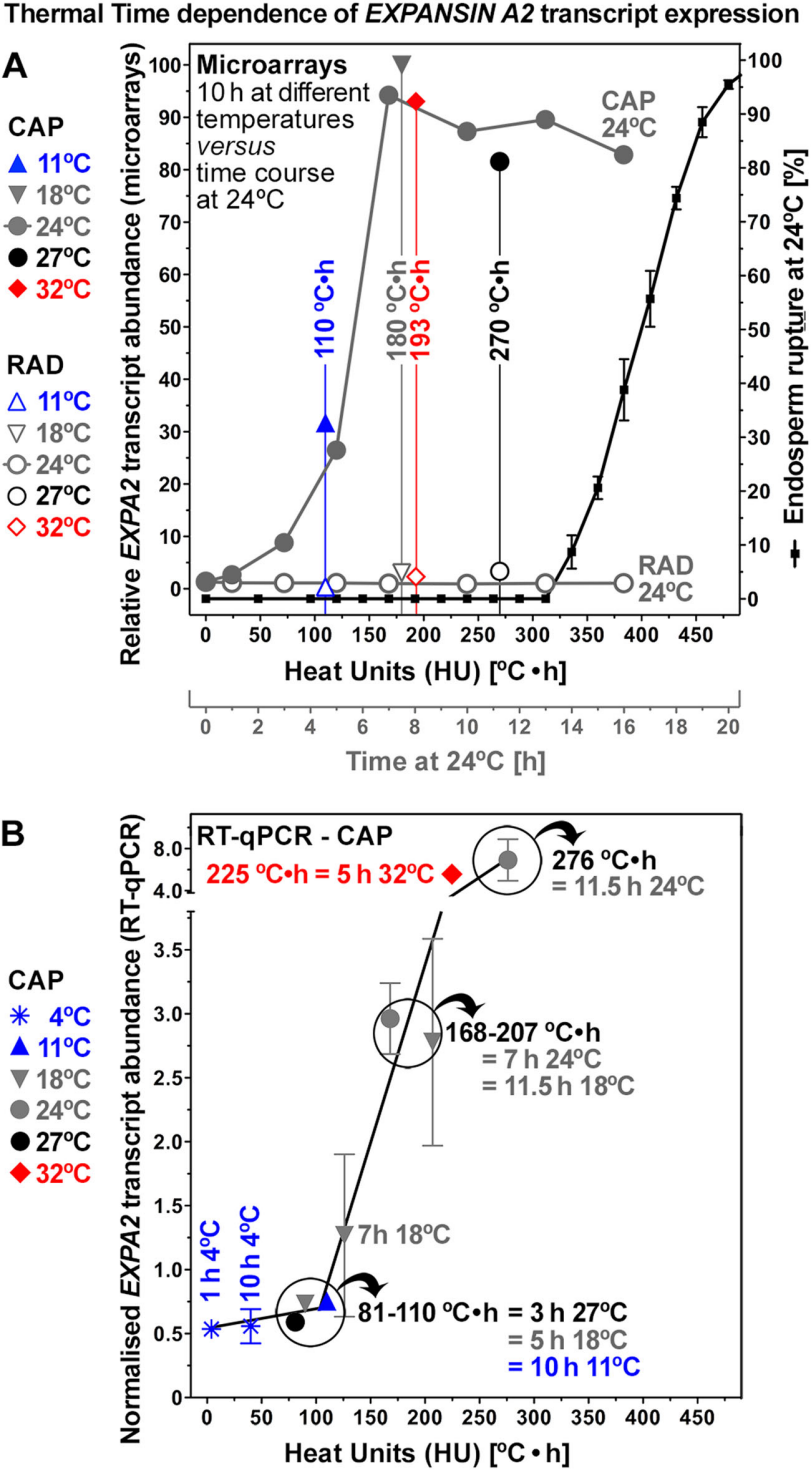
Thermal‐time dependence of *EXPANSIN A2* gene expression during *Lepidium sativum* CAP weakening and seed germination. (A) Relative transcript abundances of *LesaEXPA2* in the CAP (micropylar endosperm) and RAD (radicle and lower hypocotyl) and seed germination presented along a heat unit (HU in °C•h, accumulated temperature over time above *T*_b_ = 0°C) scale *x*‐axis. The corresponding times for the time course transcriptomes at 24°C are shown below the *x*‐axis. Relative mean ± SEM values (*N* = 4). (B) RT‐qPCR expression analysis of *LesaEXPA2* in the CAP along a HU scale *x*‐axis. Seeds were imbibed at different time‐temperature combinations, as indicated in the graph, to generate the specified HU values. Relative mean ± SEM values (*N* = 4). Note that the RT‐qPCR quantified *LesaEXPA2* transcript abundances increased in a roughly linear and thermal‐time compliant manner above a threshold HU value of ~80°C•h in the sub‐optimal temperature range to a plateau reached at ~270°C•h (corresponding to the optimal temperature region).

The α‐expansin gene *EXPANSIN A2 (LesaEXPA2)* was expressed in an endosperm‐specific manner during *L. sativum* seed germination (Figure 5)*. LesaEXPA2* is an UP‐regulated CAP DEG in both the time course and in the temperature transcriptomes (Figure 4A). It is the only expansin DEG identified in the CAP temperature transcriptomes, with other expansins identified as CAP DEGs in the time course transcriptomes (Figure S2). *LesaEXPA2* exhibits type‐2 expression pattern as it is downregulated by colder temperature and its transcript abundances are lower at smaller HU values in the microarrays (Figure 5A) and in the RT‐qPCR analysis (Figure 5B). In the sub‐optimal and optimal temperature range, between ~80°C·h and ~270°C·h, *LesaEXPA2* transcript abundances showed a linear relationship with HU values, indicative for a thermal‐time compliant gene expression pattern. Above ~270°C·h in the time course transcriptomes where CAP weakening progresses, and in the supra‐optimal temperature range at 32°C (~225°C·h) where CAP weakening was inhibited (Figure 2), *LesaEXPA2* transcript abundances remained roughly constant at a high level (Figure 5). In agreement with a causal role of LesaEXPA2 in CAP weakening, reduced *LesaEXPA2* expression inhibited CAP weakening and ER were observed at 24°C in transgenic *L. sativum* seeds over‐expressing the *DELAY OF GERMINATION 1 (DOG1)* gene (Figure S3 and Graeber et al. 2014). *LesaEXPA2* therefore seems required for CAP weakening and is expressed in a thermal‐time compliant manner in the sub‐optimal temperature range, but it alone is not sufficient to confer CAP weakening and germination (Figures 5 and S3).

**Figure 6:**
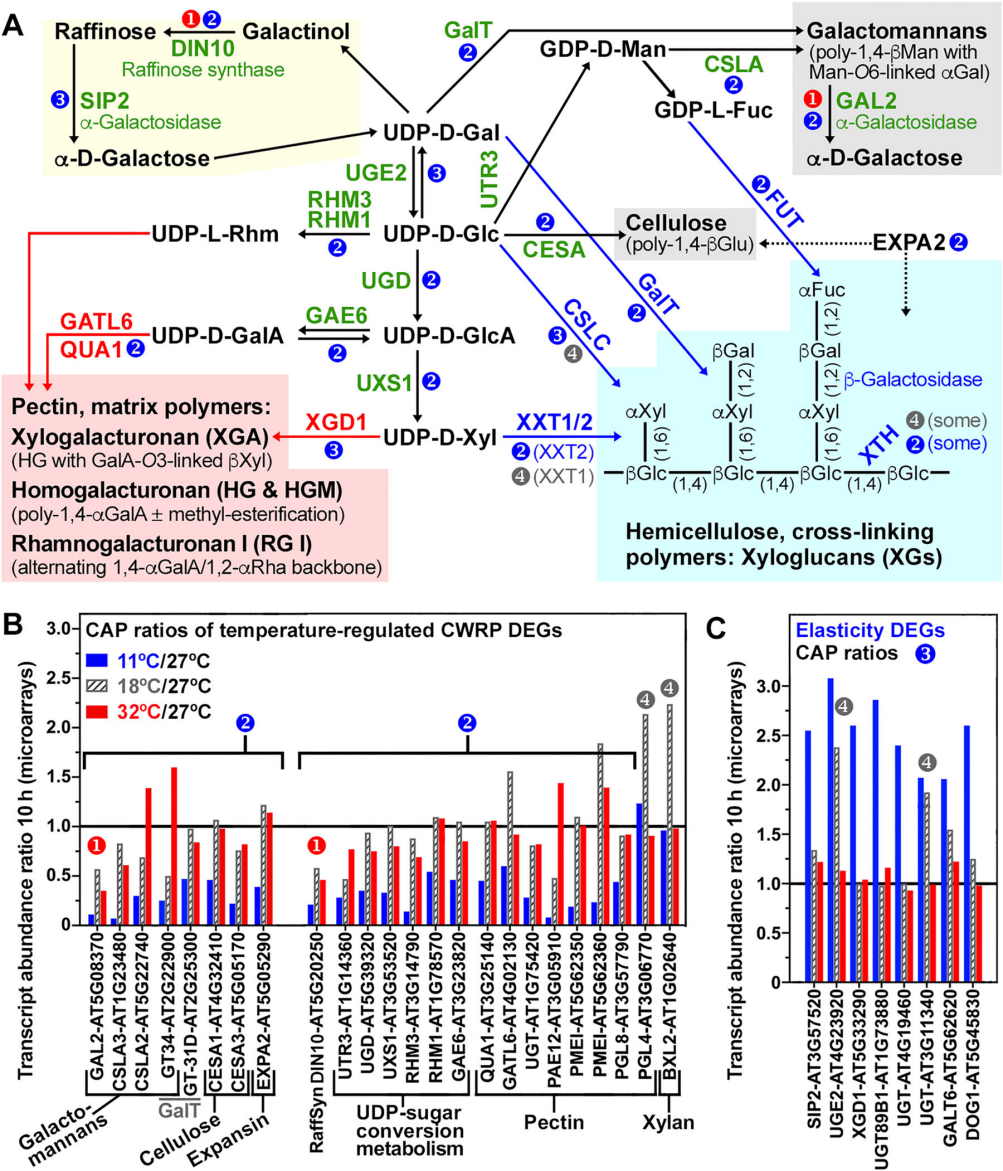
Cell wall metabolism and cell wall remodelling protein (CWRP) gene expression during *Lepidium sativum* micropylar endosperm (CAP) weakening. (A) Summary scheme of CAP cell wall metabolism depicting major cell wall polysaccharide components (cellulose, hemicelluloses, pectins, galactomannans), the raffinose cycle and connecting UDP‐sugar metabolism, as well as identified CAP CWRP DEGs and their type of expression (*numbers, see* Figure 4 *for details*). An example structure of xyloglucan (XG) hemicellulose and key XG‐biosynthesis and XG‐modifying enzymes are indicated. (B) Expression ratios in the temperature transcriptomes at 10 h compared to the optimal temperature (27°C) of CAP CWRP DEGs identified (transcript abundance ratios ≥ 2 or ≤ 0.5) CAP weakening gene candidates. Numbers indicate type of expression: type‐1 (down at 32°C), type‐2 (down at 11°C), type‐4 (up at 18°C). (C) Expression ratios in the temperature transcriptomes of CAP CWRP DEGs identified as type‐3 (up at 11°C) stiffening/elasticity genes. Abbreviations (*panels A–C*): α‐d‐galactose (d‐Gal), d‐glucose (d‐Glc), d‐glucuronic acid (d‐GlcA), d‐xylose (d‐Xyl), d‐galacturonic acid (d‐GalA), l‐rhamnose (l‐Rham), d‐mannose (d‐Man), cellulose synthase (CESA), xyloglucan (XG) xylosyltransferase (XXT), cellulose synthase like‐C (CSLC), α‐fucosyltransferase (FUT), XG endotransglycolase/hydrolase (XTH), α‐expansin (EXPA), cellulose synthase like‐A (CSLA), α‐galactosidase (GAL2, SIP2 (SEED IMBIBITION 2)), galacturonosyltransferase (GAUT, GalT, QUA1 (QUASIMODO 1)), galacturonosyltransferase‐like (GATL), xylogalacturonan (XGA) xylosyltransferase (XGD1 (XGA DEFICIENT 1)), UDP‐glucose dehydrogenase (UGD), UDP‐d‐glucuronate 4‐epimerase (GAE), UDP‐d‐glucose 4‐epimerase (UGE), UDP‐glucuronic acid decarboxylase (UXS; enzymatic product UDP‐xylose), UPD‐galactose transporter (UTR), rhamnose biosynthesis (RHM), pectinacetylesterase (PAE), pectin methylesterase inhibitor (PMEI), pectin lyase‐like/polygalacturonase‐like (PGL), β‐xylosidase (BXL), UDP‐glycosyltransferase (UGT), hydroxyproline O‐galactosyltransferase (GALT), raffinose synthase 6 (DIN10 (DARK INDUCIBLE 10)), DELAY OF GERMINATION 1 (DOG1).

The identified lists of upregulated CAP CWRP DEGs (Figure 4, SI Datasets S12 and 13) suggested that cellulose, galactomannans, xyloglucans, xylans, pectins, as well as raffinose and the connecting UDP‐sugar metabolism are targets for cell wall remodelling (Figure 6A). To categorize the identified candidate CAP weakening and stiffness DEGs based on their expression patterns (type‐1,‐2,‐3,‐4, see Figure 4C), we initially calculated the transcript abundance ratios for 11°C, 18°C and 32°C compared to *T*_o_ (27°C) (Figure 6B,C). As *LesaEXPA2*, most CAP DEGs had type‐2 (down at 11°C) expression pattern (Figure 6B), and this includes genes encoding enzymes targeting cellulose and galactomannan (Figures 6 and S4). Among them are two cellulose synthase (*CESA*) involved in cellulose biosynthesis, and two cellulose synthase‐like (*CSLA*), involved in 1,4‐β‐mannan backbone biosynthesis (Amos and Mohnen 2019; Daras et al. 2021; Voiniciuc et al. 2015). To investigate if these type‐2 DEGs are regulated in a thermal‐time compliant manner in the sub‐optimal temperature range, we used RT‐qPCR to test if identical HU values (270°C·h) generated by different temperature–time combinations (11°C + 25 h, 18°C + 15 h, 27°C + 10 h) resulted in equal CAP transcript abundances for *LesaCSLA2*, *LesaCESA1* and *LesaCESA3*. This was however not the case, their transcript abundances were significantly lower at the 11°C–25 h combination representing 270°C·h (Figure S4). We conclude from this, that these type‐2 CWRP CAP DEGs are not regulated in a thermal‐time compliant manner over the entire suboptimal temperature range and that there is a temperature‐specific response to chilling (11°C).

**Figure 7:**
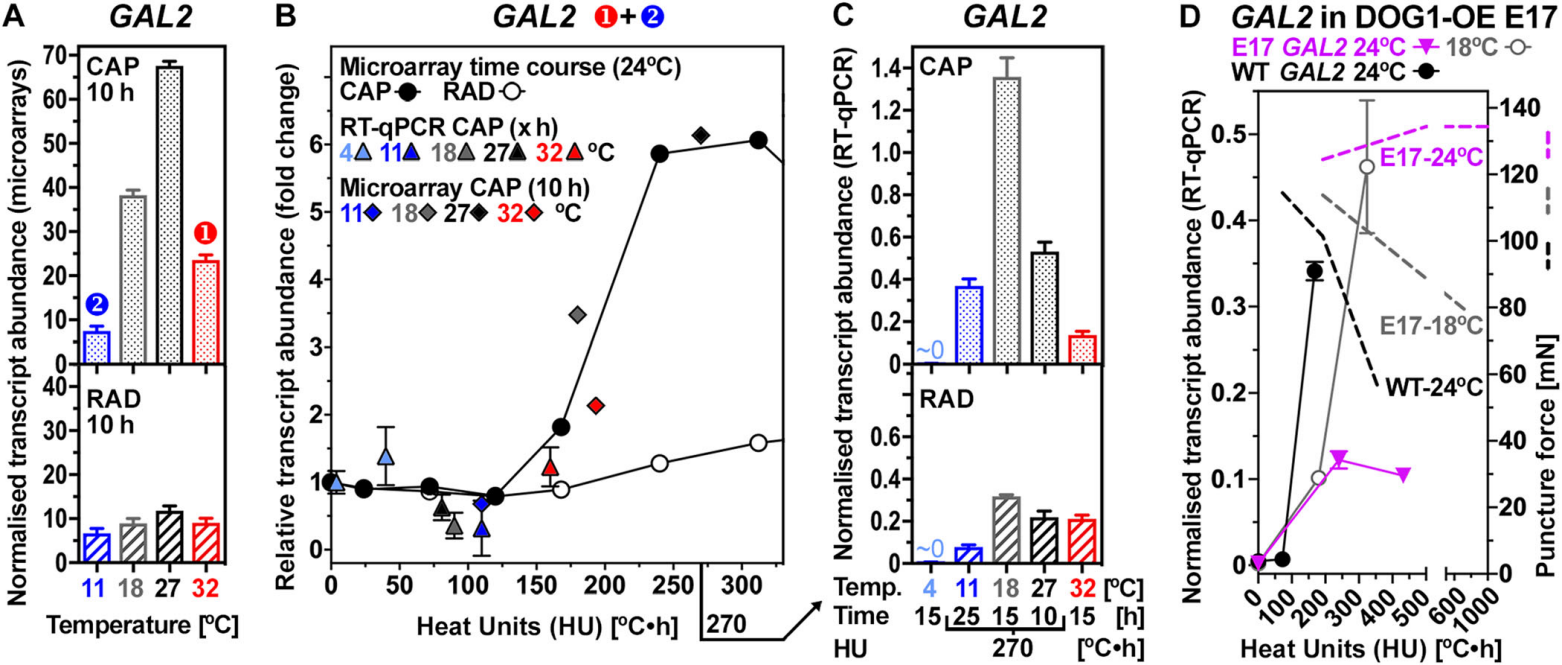
Temperature and DOG1 regulation of galactomannan‐degrading α‐galactosidase *LesaGAL2* expression during *Lepidium sativum* seed germination. (A) *LesaGAL2* transcript abundances in the CAP (micropylar endosperm) and RAD (radicle and lower hypocotyl) in the temperature transcriptomes at 10 h at the temperatures indicated. Mean ± SEM values (*N* = 4). Note that *LesaGAL2* was identified as a type‐1 (down at 32°C) and type‐2 (down at 11°C) CAP CWRP DEG. (B) *LesaGAL2* transcript abundances in the CAP and RAD in the time course (microarrays at 24°C) and temperature (microarrays at 10 h) transcriptomes and expression verification by RT‐qPCR (independent experiment) presented along a heat unit (HU in °C•h above *T*_b_ = 0°C) scale *x*‐axis. Mean ± SEM values (*N *= 4). The 270°C•h HU value is indicated as it was used to conduct RT‐qPCR analysis to test for thermal‐time expression (*see panel C*). (C) RT‐qPCR analysis of *LesaGAL2* transcript abundances at 15 h (4°C, 18°C, 32°C) and at an identical HU value (270°C•h) generated by different time‐temperature combinations as indicated. Mean ± SEM values (*N* = 4). The *LesaGAL2* transcript abundances differed in the CAP at 270°C•h in the sub‐optimal temperature range indicating that *LesaGAL2* is not expressed in a thermal‐time compliant manner. (D) Expression analysis of *LesaGAL2* in DOG1‐overexpressing transgenic *L. sativum* E17 seeds presented along a HU scale *x*‐axis. Note that CAP weakening is blocked (see puncture force values indicated in the graph and presented in detail in Figure S3) and germination inhibited is blocked in E17 seeds imbibed at 24°C, the *T*_o_ of wild type (WT) seeds (Graeber et al. 2014). Mean ± SEM values (*N* = 4).

Although in Brassicaceae seeds galactomannan has so far only been detected in mucilage, there is considerable evidence that galactomannan‐hydrolysing enzymes are also involved in Brassicaceae endosperm weakening and rupture (Carrillo‐Barral et al. 2018; Feurtado et al. 2001; Lee et al. 2012; Rodríguez‐Gacio et al. 2012). Beyond seed germination, α‐galactosidases are involved in the fruit tissue softening and *A. thaliana* mutants demonstrated that cell wall localised *α‐GALACTOSIDASE 2* (*GAL2*) is involved in cell expansion growth (Chrost et al. 2006). In *L. sativum* seeds, *LesaGAL2* is an upregulated CAP DEG in the time course and the temperature transcriptomes (Figures 4A and 6). As *LesaEXPA2*, also *LesaGAL2* was expressed in a CAP‐specific manner (Figure 7). The *LesaGAL2* transcript abundances were downregulated by sub‐optimal temperatures (strong at 11°C, weaker at 18°C), indicative for a type‐2 weakening gene, and were also downregulated by supra‐optimal temperature (32°C), indicative for a type‐1 weakening gene (Figure 7A). Because reduced expression was associated with the inhibition of CAP weakening at nonoptimal colder and warmer temperatures, *LesaGAL2* could therefore be essentially required for CAP weakening at sub‐optimal and supra‐optimal temperatures (Figure 2). However, *LesaGAL2* transcript abundances quantified at an identical HU value (270 C·h) generated by different time‐temperature combinations, differed especially for the 18°C–15 h combination (Figure 7C). This demonstrates that *LesaGAL2* expression is not regulated in a thermal‐time compliant manner over the entire sub‐optimal temperature range, but rather exhibits a specific response to the 18°C temperature.

The *DOG1* gene determines the permissive temperature window for germination by regulating the expression of CAP weakening genes (Graeber et al. 2014). To further investigate the role of *LesaGAL2* we utilised *L. sativum* DOG1‐overexpressing lines (E17, B13) for which we have demonstrated earlier that the DOG1‐mediated inhibition of seed germination at 24°C (*T*_o_ of wild type, WT) was solely due to the inhibition of CAP weakening; the embryo growth potential was not affected by the DOG1 overexpression (Graeber et al. 2014). Overexpression of DOG1 in transgenic E17 seeds resulted in severely delayed germination at WT *T*_o_ (24°C): 50% ER was at ~350°C·h (15 h) for WT versus ~15,000°C·h (> 600 h) for E17 and the decrease in CAP puncture force was completely inhibited for E17 at 24°C (Figures 7D and S3). In agreement with a causal role of LesaGAL2 in CAP weakening, a severe reduction in *LesaGAL2* transcript accumulation was observed in E17 and B13 seeds (Figures 7D and S3). This strongly suggests that the DOG1‐induced temperature‐dependent mechanism repressed *LesaGAL2* and *LesaEXPA2* at 24°C (Figure S3). This is further supported by the fact that E17 and WT seed germination was similar at 18°C, as was the CAP weakening and WT‐like accumulation of *LesaGAL2* transcripts (Figures 7D and S3). These findings suggest that in *L. sativum* seeds DOG1 controls the germination temperature responses by regulating the expression of downstream genes including *LesaGAL2* and *LesaEXPA2*. In agreement with this, *LesaDOG1* is itself a DEGs in the temperature arrays with specifically higher transcript abundances at 11°C, even when compared at similar HU values (Figures 6C and S5).

### 3.4 Temperature Regulation of DEGs Involved in UDP‐Sugar Metabolism, Pectin and Xyloglucan Remodelling and Cold‐Induced CAP Elasticity

**Figure 8:**
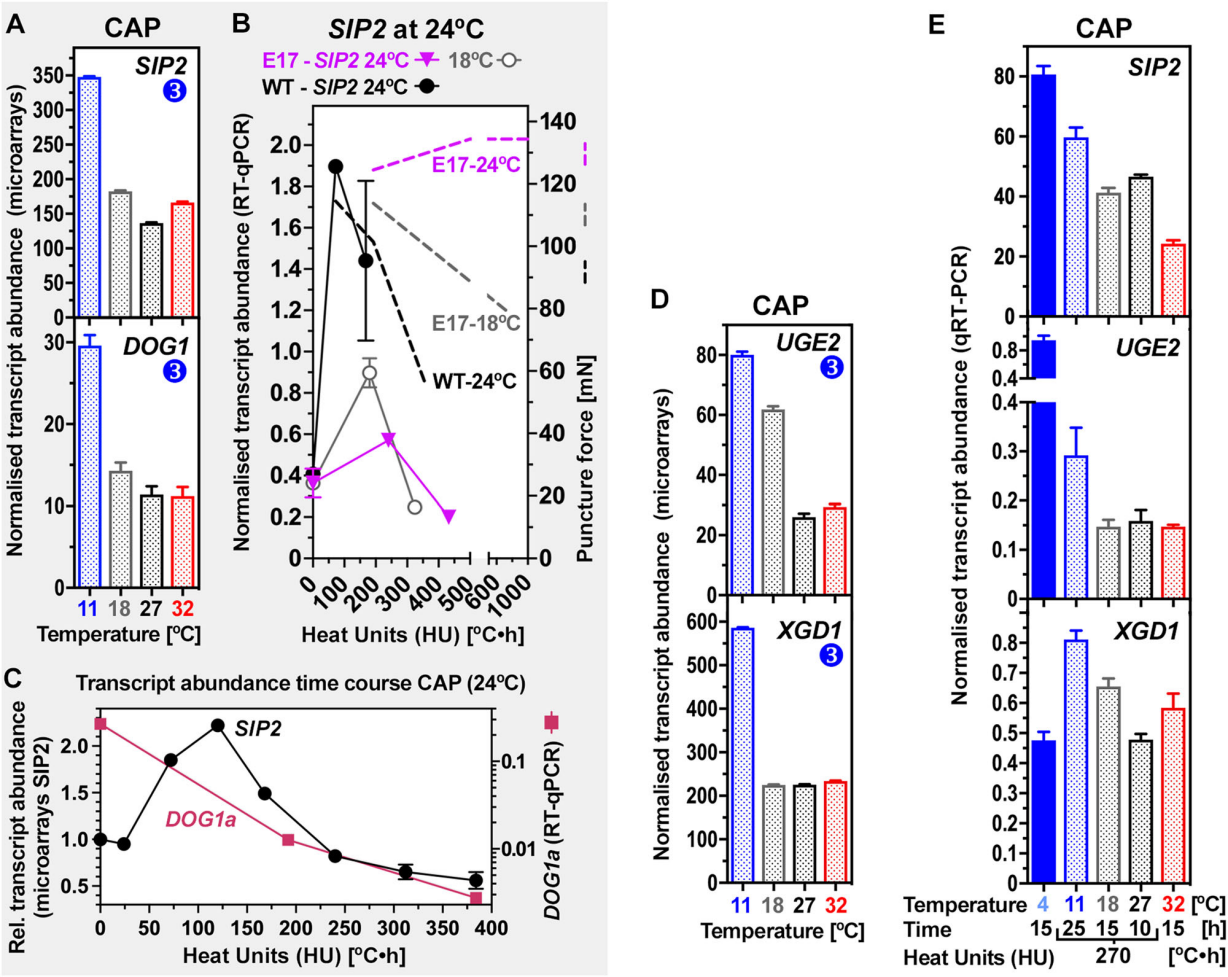
Temperature and DOG1 regulation of raffinose‐degrading α‐galactosidase *LesaSIP2* (*SEED IMBIBITION 2*), UDP‐sugar metabolism and pectin‐related gene expression during *Lepidium sativum* seed germination. (A) *LesaSIP2* and *LesaDOG1* transcript abundances in the CAP (micropylar endosperm) in the temperature transcriptomes (microarrays) at 10 h at the temperatures indicated. Mean ± SEM values (*N* = 4). Note that *LesaSIP2* and *LesaDOG1* were identified as a type‐3 (up at 11°C) CAP stiffening/elasticity DEGs. (B) Expression analysis of *LesaSIP2* and puncture force analysis in DOG1‐overexpressing transgenic *L. sativum* E17 seeds presented along a heat unit (HU in °C•h above *T*_b_ = 0°C) *x*‐axis. Mean ± SEM values (*N *= 4). (C) *LesaSIP2* (time course microarrays) and *LesaDOG1a* (RT‐qPCR) transcript abundances in the CAP presented along a presented along a HU scale *x*‐axis. Mean ± SEM values (*N* = 4). For further expression analyses of *LesaSIP2* and *LesaDOG1* see Figure S5. (D) CAP transcript abundances of UDP‐sugar metabolism *LesaUGE2* and pectin‐related Lesa*XGD1* DEGs in the temperature transcriptomes (microarrays) at 10 h at the temperatures indicated. Mean ± SEM values (*N* = 4). Abbreviations: XGA, xylogalacturonan; XGD1, xylosyltransferase XGA DEFICIENT 1; UGE, UDP‐d‐glucose 4‐epimerase. (E) RT‐qPCR analysis of UDP‐sugar metabolism and pectin‐related gene expression in the CAP at 15 h (4°C, 18°C, 32°C) and at an HU value of 270°C•h HU value generated by different time‐temperature combinations as indicated. Mean ± SEM values (*N* = 4). Their transcript abundances differed in the CAP at 270°C•h indicating that these genes are not expressed in a thermal‐time compliant manner. For further expression analyses see Figures S6 and S7.

Genes encoding alkaline α‐galactosidase *SEED IMBIBITION 2* (*SIP2*), UDP‐glucose 4‐epimerase *UGE2*, three UDP‐glycosyltransferases (UGTs), galactosyltransferase *GATL6*, and xylogalacturonan (XGA) xylosyltransferase *XGA DEFICIENT 1* (*XGD1*) were identified as *L. sativum* UP‐regulated CAP DEGs with type‐3 expression patterns, they were specifically induced by chilling at 11°C (Figure 6C). The increase in CAP elasticity at 11°C (Figure 2) was associated with decreased raffinose synthase *LesaDIN10* (Figure 6B, type‐2 and type‐1 pattern) and increased transcript abundances of *LesaSIP2* (Figure 6C, type‐3 pattern), suggesting α‐d‐galactose (d‐Gal) release from raffinose. *LesaSIP2* expression was specifically induced in the CAP at 11°C and 4°C where also *LesaDOG1* transcript abundance was high (Figures 8A–C and S5). RT‐qPCR analyses at equal HU values showed that neither *LesaSIP2*, *LesaDIN10* nor *LesaDOG1* were regulated in a thermal‐time compliant manner in the CAP. As for *LesaGAL2* and *LesaEXPA2*, DOG1‐overexpression in E17 and B13 seeds caused a severe reduction in *LesaSIP2* transcript accumulation (Figures 8B and S3). In *A. thaliana* SIP2 is known for its substrate specificity for raffinose (Peters et al. 2010) and SIP2‐mediated release of d‐Gal is known to be required for rapid seed germination (Gangl and Tenhaken 2016). The released d‐Gal would be converted via UDP‐d‐galactose to UDP‐d‐glucose by UDP‐d‐glucose 4‐epimerase (UGE) and from there into other UDP‐sugars (Figure 6A). UGE2 is known for its specific roles in cell wall biosynthesis and cell growth (Hou et al. 2021; Rösti et al. 2007; Temple et al. 2016). The *LesaUGE2* gene was identified as a type‐3 DEG induced in the *L. sativum* CAP by the cold‐treatment (Figure 6C, 8, S6). RT‐qPCR analyses of *LesaSIP2* and *LesaUGE2* CAP transcript abundances at equal HU values (270°C·h) generated by different temperature–time combinations demonstrated that they are not regulated in a thermal‐time compliant manner but is specifically induced by cold temperatures (Figure 8E). DEGs encoding other enzymes involved in the UDP‐sugar conversion metabolism exhibited type‐2 expression pattern, and they were also not regulated in a thermal‐time compliant manner (Figures 6B and S6). Taken together this strongly suggest that d‐Gal release and conversion via UDP‐sugars (Figure 6A) are key process specifically important for cell wall remodelling leading to the observed increase in CAP tissue elasticity at 11°C (Figure 2).

Conserved properties in the endosperm cell wall composition of *L. sativum* and *A. thaliana* include cellulose, xyloglucan and pectins (Lee et al. 2012; Levesque‐Tremblay et al. 2015; Scheler et al. 2015). For XGA pectin biosynthesis, *LesaXGD1* was identified as a type‐3 DEG specifically induced in the *L. sativum* CAP by the cold‐treatment (Figures 6, 8D, and S7). Pectin‐targeting CAP DEGs are known from *A. thaliana* to be involved in hypocotyl stiffness, cell adhesion/separation and seed mucilage expansion (Burgert and Keplinger 2013; Verger et al. 2018). RT‐qPCR verification of the CAP transcript abundances of the *LesaXDA1* and other pectin‐related genes at an identical HU value (270°C·h), generated by different time‐temperature combinations, demonstrated that these DEGs were not regulated in a thermal‐time compliant manner, but were specifically induced by 11°C (Figures 6C, 8E, and S7).

**Figure 9:**
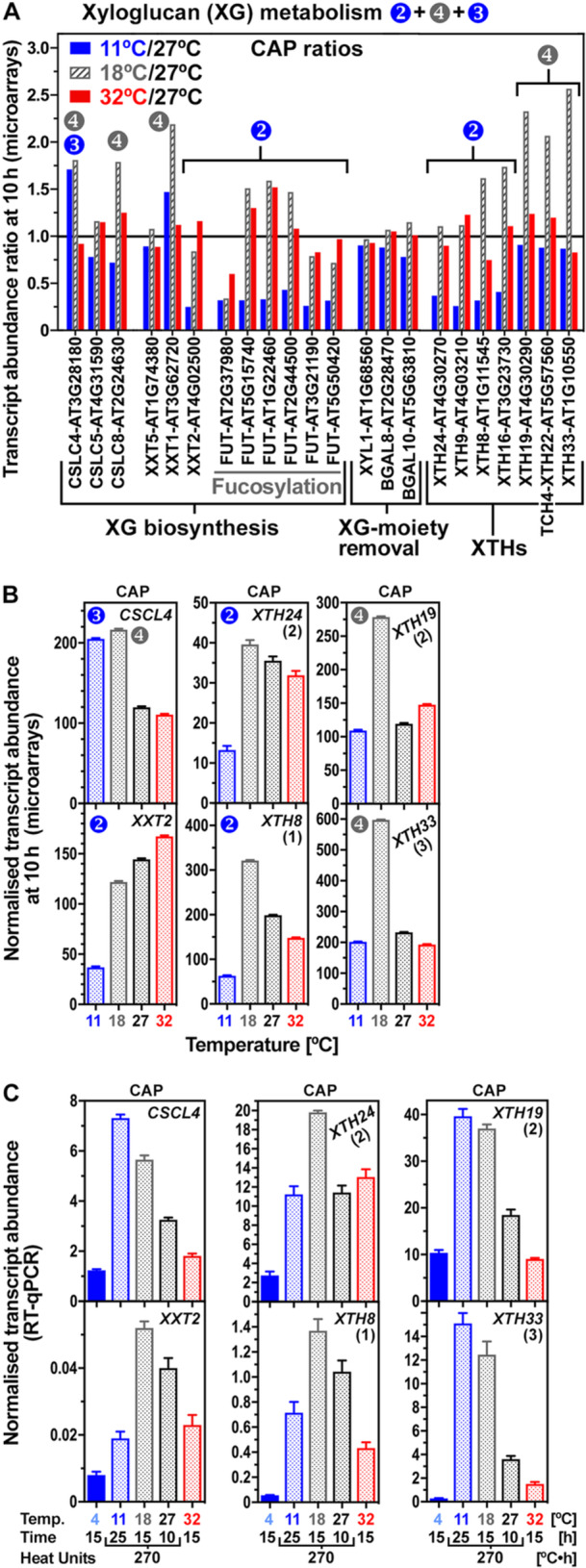
Temperature regulation of genes encoding xyloglucan (XG) biosynthesis and modification enzymes during *Lepidium sativum* micropylar endosperm (CAP) weakening. (A) Expression ratios in the temperature transcriptomes at 10 h compared to the optimal temperature (27°C) of identified XG‐related CAP DEGs (transcript abundance ratios ≥ 2 or ≤ 0.5). Numbers indicate their type of expression: type‐2 (down at 11°C), type‐3 (up at 18°C, stiffening/elasticity genes) type‐4 (up at 18°C). Note that assignment of type‐3 and/or type‐4 expression patterns for *LesaCSCL4* and *LesaCSCL8* was based on a > 1.7‐fold ratio. Abbreviations: BGAL, β‐galactosidase; CSLC, cellulose synthase‐like‐C; FUT, α‐fucosyltransferase; XTH, XG endotransglycolase/hydrolase; XXT, XG xylosyltransferase; XYL1, α‐xylosidase. (B) XG‐related CAP DEG transcript abundances in the temperature transcriptomes (microarrays) at 10 h at the temperatures indicated. Mean ± SEM values (*N* = 4). Numbers indicate their type of expression: type‐2 (down at 11°C), type‐3 (up at 11°C, stiffening/elasticity gene), type‐4 (up at 18°C). (C) RT‐qPCR analysis of XG‐related gene expression in the CAP at 15 h (4°C, 18°C, 32°C) and at an identical HU value (270°C•h) generated by different time–temperature combinations as indicated. Mean ± SEM values (*N* = 4). The transcript abundances differed in the CAP at 270°C•h indicating that these genes are not expressed in a thermal‐time compliant manner. For further expression analyses see Figures S8 and S9.

Xyloglucan (XG) deficiency leads to a reduction in cell wall stiffness, altered pectin level and cellulose bundling (Burgert and Keplinger 2013; Bou Daher et al. 2024; Kim et al. 2020; Park and Cosgrove 2012; Sowinski et al. 2022; Xiao et al. 2016). Figures 6 and 9 show that CAP DEGs encoding enzymes involved in pectin, xylan and XG biosynthesis and/or modification either exhibited type‐2 (lower at 11°C), type 3 (higher at 11°C) or type‐4 (higher specifically at 18°C) transcript abundance patterns. Compared to the optimal temperature, type‐4 DEGs are specifically upregulated at 18°C, and usually not at 11°C where CAP elasticity was increased (Figures 2 and 4). Among the XG targeting CAP DEGs, a XG xylosyltransferase (*LesaXXT1*), two cellulose synthase like‐C (CSLC) and three xyloglucan endotransglycolase/hydrolase (XTH) genes exhibit type‐4 expression patterns (Figures 9, S8 and S9). Roles in CAP weakening were proposed for XTH genes with endosperm‐specific expression (Chen et al. 2002; Endo et al. 2012; Zhang et al. 2024a). The general pattern in the *L. sativum* CAP was XTH transcript accumulation during the germination time course (Figure S10), and in the temperature arrays specific XTH DEGs had either type‐2 or type‐4 expression patterns (Figures 9 and S9). RT‐qPCR verification of its expression at an equal HU value (270°C·h) generated by different temperature–time combinations however demonstrated that none of the XTH, XXT and CSLC CAP DEGs had a fully thermal‐time compliant expression pattern over the entire sub‐optimal temperature range (Figure 9).

## 4 Discussion

Weakening of the micropylar endosperm (CAP) covering the radicle and increasing embryo growth potential (RAD) interact to control the completion of seed germination by ER (Bewley 1997; Finch‐Savage and Leubner‐Metzger 2006; Graeber et al. 2014; Hourston et al. 2022; Linkies et al. 2009; Müller et al. 2009; Nonogaki 2019; Scheler et al. 2015; Steinbrecher and Leubner‐Metzger 2017; Yan et al. 2014b). Endosperm weakening is confined to the CAP region and includes thinning and/or loosening of cell walls, cell separation (loss of cell‐cell adhesion at the pectin‐rich middle lamellae), tissue autolysis and/or hole formation, and cell vacuolation (Bethke et al. 2007; Endo et al. 2012; Linkies et al. 2009; Morris et al. 2011; Müller et al. 2006). Abscisic acid (ABA) inhibits *L. sativum* CAP weakening and this is associated with linear relationship between CAP tissue strength (puncture‐force) and ER (Linkies et al. 2009). While we obtained a linear relationships between temperature and *L. sativum* germination speed in the sub‐optimal (colder, < *T*_o_) and supra‐optimal (warmer, > *T*_o_) temperature ranges (Figure 1), the underpinning biomechanical mechanisms differed between temperatures within the sub‐optimal range itself, as well as between sub‐ and supra‐optimal temperatures (Figure 2). Direct biomechanical quantification revealed that temperatures alter the mechanical CAP properties in a distinct and temperature‐specific manner. Within the sub‐optimal temperature range CAP stiffness was not appreciably affected by the slightly colder (18°C) and not at all by optimal temperatures (24°–27°C), while CAP tissue strength (puncture‐force) decreased with time in association with TR and ER. A similar roughly twofold reduction in germination rates by heat (32°C) and chilling (11°C) temperature stress compared to the optimal temperature (*T*_o_) was observed. Heat completely blocked the reduction in CAP tissue strength without affecting CAP stiffness. In contrast to this, chilling caused a moderate reduction in CAP tissue strength combined with a reduction in CAP stiffness. The underpinning biomechanical mechanisms of CAP weakening therefore differ fundamentally between the optimal, supra‐optimal (heat) and sub‐optimal (slightly colder vs. chilling) temperatures. In addition to CAP tissue strength (puncture force) changes (Steinbrecher and Leubner‐Metzger 2017), CAP stiffness changes were therefore discovered as a new biomechanical hallmark of endosperm CAP weakening.

It is known that nonoptimal temperatures and other abiotic stress factors affect seedling growth through CWRP‐mediated cell wall remodelling which changes the hypocotyl's biomechanical properties (Burgert and Keplinger 2013; Le Gall et al. 2015; Tenhaken 2015). Depending on the abiotic stress factor different biochemical cell wall changes and different biomechanical mechanisms are employed by targeting cellulose, hemicellulose, pectin and other cell wall components. *Arabidopsis thaliana* mutants with severe reduction in the pectin homogalacturonan (*qua2*, *mur1*) for example are largely affected in stiffness but only marginally in strength of their hypocotyl cell walls (Abasolo et al. 2009; Burgert and Keplinger 2013). In contrast to this, hypocotyls with an altered xyloglucan (XG) structure or reduced XG contents (*xxt1/xxt2*, *cslc*, *mur3*) showed a loss in both stiffness and strength (Burgert and Keplinger 2013; Bou Daher et al. 2024; Park and Cosgrove 2012; Sowinski et al. 2022; Xiao et al. 2016). By using comparative transcriptome analysis across different temperatures and imbibition times we identified CWRP DEGs in the *L. sativum* CAP associated with the observed changes in CAP puncture force and/or stiffness (Figures 3, 4, 5, 6, 7, 8, 9). CWRP candidates to confer the chilling‐induced reduction in CAP stiffness include the *SIP2*, *UGE2* and *XGD1* DEGs which were specifically upregulated at 11°C (type‐3 expression pattern, Figure 6C). This suggests that d‐Gal released from raffinose by the α‐galactosidase SIP2 (Gangl and Tenhaken 2016; Peters et al. 2010) is, via the UDP‐sugar metabolism involving UGE2 (Hou et al. 2021; Rösti et al. 2007; Temple et al. 2016), utilised by XGD1 to alter pectic xylogalacturonan (XGA) (Figure 6A). The *L. sativum* CAP is rich in XGA and XGD1 is known to be specifically involved in XGA biosynthesis (Amos and Mohnen 2019; Jensen et al. 2008; Lee et al. 2012). Chilling‐induced upregulation of several UGTs and downregulation of QUA1 and other pectin‐related enzymes (Figure 6) may also contribute to the CAP stiffness reduction at 11°C. Pectin alterations in the *A. thaliana qua1* and other mutants cause altered hypocotyl cell wall stiffness, reduced cell‐cell adhesion (Abasolo et al. 2009; Burgert and Keplinger 2013; Verger et al. 2018) and altered seed germination (Daher and Braybrook 2015; Lee et al. 2012; Levesque‐Tremblay et al. 2015; Müller et al. 2013; Scheler et al. 2015).

The identified type‐2 CAP CWRP DEGs were instrumental in addressing the fundamental question if CWRP gene expression is regulated in a thermal‐time compliant manner to provide the observed linear relationship between temperature and germination rates (speed). Addressing this question was achieved by comparison of their transcript abundances in the temperature (at 10 h) and time course (at 24°C) transcriptomes at corresponding heat unit values (HU, in °C·h) above *T*_b_ (0°C), and by RT‐qPCR verification at equal HU values generated by different temperature–time combinations. This approach identified that the transcript abundances of *LesaEXPA2* increased in a linear manner above a threshold value of ~80°C·h until ~270°C·h which roughly covers the entire sub‐optimal temperature range (Figure 5). Expression of the *EXPA2* genes in *L. sativum* and *A. thaliana* is endosperm‐specific and *EXPA2* is known to promote gibberellin‐induced CAP weakening and seed germination (this study; Sánchez‐Montesino et al. 2019; Scheler et al. 2015; Yan et al. 2014a). Expansins are required for plant cell growth by targeting the biomechanical hotspots (load‐bearing cellulose‐xyloglucan‐cellulose junctions) to mediate cell wall loosening required for water uptake and irreversible plastic cell expansion (Cosgrove 2016, 2018; Steinbrecher and Leubner‐Metzger 2017). Expansin‐mediated cell wall loosening further contributes to cell wall remodelling by allowing cell wall hydrolases to access their polysaccharide substrates (Samalova et al. 2022; Zhang et al. 2019). The expression of α‐expansin genes including *EXPA2* and *EXPA9* is not appreciably affected by ABA but downregulated by DOG1 (this study; Chen and Bradford 2000; Graeber et al. 2014; Voegele et al. 2011; Weitbrecht et al. 2011; Xue et al. 2021). The *LesaEXPA2* expression patterns therefore neither explain the ABA‐sensitivity of CAP weakening nor its inhibition at hot temperatures (Figure 2). In conclusion, *LesaEXPA2* contributes to the linear relationship between temperature and germination speed in the sub‐optimal to optimal temperature range. It is however not the sole rate‐limiting CWRP in all conditions and is known to in addition to act by making cell wall polysaccharides accessible to a battery of cell‐wall hydrolases.

A linear regulation of enzymatic activities by temperature has been proposed for CAP weakening rather than a single, rate‐limiting enzyme (Bewley 1997; Dahal et al. 1997; Finch‐Savage and Leubner‐Metzger 2006; Hegarty 1973; Trudgill et al. 2005). The α‐galactosidase *LesaGAL2* CAP DEG had type‐2 (reduced at 11°C) and type‐1 (reduced at 32°C) expression pattern (Figures 6 and 7). RT‐qPCR verification of its expression at an equal HU value (270°C·h) generated by different temperature–time combinations however demonstrated that its expression pattern is not fully thermal‐time compliant over the entire sub‐optimal temperature range, it is specifically upregulated at 18°C. Although *GAL2* may be required for galactomannan degradation and CAP weakening, it alone is not sufficient and requires the concerted action of other CWRPs. Although not identified as > 2‐fold upregulated CAP DEGs in the microarrays, accumulation of endo‐β‐1,4‐mannanase (*MAN*) gene expression and enzyme activity have been demonstrated in the *L. sativum* CAP (Morris et al. 2011), and *MAN* transcript accumulation as well as *MAN*‐related germination phenotypes were observed in other Brassicaceae (Carrillo‐Barral et al. 2018; Rodríguez‐Gacio et al. 2012). Dahal et al. (1997) demonstrated that endo‐β‐1,4‐mannanase enzyme activity increased in tomato seeds in accordance with accumulated thermal‐time over the sub‐optimal temperature range (10°C–25°C), but the activities did not correlate with the effects of reduced water potential or ABA on seed germination. Tomato CAP weakening is biphasic with galactomannan degradation being achieved in the first ABA‐insensitive step, but only the second ABA‐controlled step leads to ER (Bewley 1997; Feurtado et al. 2001; Rodríguez‐Gacio et al. 2012; Toorop et al. 2000). Caveats for correlating in vitro measured enzyme activities with thermal‐time models include that enzyme activity itself are temperature‐dependent (Parent et al. 2010). Caveats for using cumulative transcript abundances as proxy for CRWP activities (Figure S10) include that transcription and mRNA decay are subject to both passive and active rate changes by temperature (Chen et al. 2021). Results obtained using single‐seed transcriptomes revealed continuous gradients in gene expression, underpinning nonbinary variability between individual seed germination timing in a population (Krzyszton et al. 2022). Taken together, this further supports the view that not a single CWRP, but a battery of CWRPs with distinct temperature‐specific regulation on gene expression and enzyme activity level control CAP weakening and germination timing.

We found that genes encoding enzymes which target xyloglucan (XG, Figure 6) seem to be most important for *L. sativum* CAP weakening and that they represented high diversity in thermal‐time and temperature‐specific expression patterns (Figures 9 and S8‐10). XG biosynthesis CAP DEGs had either type‐2 (*XXT2*, *FUTs*; down at 11°C) or type‐4 (*XXT1*, *CSLC4*, *CSLC8*; up at 18°C) expression patterns suggesting that XG contents and fucosylation are reduced upon chilling, and that imbibition at slightly colder (18°C) may trigger XG biosynthesis. An association between reduced XG content and delayed *L. sativum* ER upon chilling is consistent with the finding that the germination of XG‐deficient *xxt1*/*xxt2 A. thaliana* mutant seeds is delayed (Lee et al. 2012). It is also known that *A. thaliana* mutants with reduced or lacking CSLC or XXT activity have hypocotyl cell walls with reduced XG contents and loss in strength (Burgert and Keplinger 2013; Bou Daher et al. 2024; Park and Cosgrove 2012; Sowinski et al. 2022; Xiao et al. 2016). XG modification by XTHs can either lead to tissue weakening or strengthening of the cell wall biomechanical properties (Holloway et al. 2021; Miedes et al. 2013; Rose et al. 2002; Takahashi et al. 2021). In *A. thaliana* XTH19 was shown to be involved in cold tolerance, hypocotyl growth and cell wall mechanics, and represents a group of XTH proteins which has only endotransglucosylase (XET) activity while other XTH proteins have in addition endohydrolytic (XEH) activity (Maris et al. 2011; Miedes et al. 2013; Rose et al. 2002; Takahashi et al. 2021). In seeds, XTH proteins including XTH19 are specifically expressed during endosperm weakening (Chen et al. 2002; Endo et al. 2012; Steinbrecher and Leubner‐Metzger 2017; Voegele et al. 2011; Zhang et al. 2024a). We identified *LesaXTH19* as a type‐4 CAP DEG with equal expression at a defined HU value in the sub‐optimal temperature range between 11°C and 18°C (Figure 9), predominant expression in the CAP, downregulated by ABA (Voegele et al. 2011) and by DOG1 (Graeber et al. 2014). The general pattern in the *L. sativum* CAP for XTH genes was transcript accumulation during the germination time course (Figures 9B and S10), and in the temperature arrays specific XTH DEGs had either type‐2 or type‐4 expression patterns (Figures 9 and S9).

Comparison of the expression patterns of the XG‐targeting XTH, CSLC, and XXT DEGs in *L. sativum* CAP tissues at an equal HU value (270°C·h) generated by distinct temperature–time combinations demonstrated that their temperature‐specific regulation is distinct from simple thermal‐time regulation (Figure 9, S8 and S9). In many cases, the type‐3 (higher at 11°C, e.g., *LesaCSCL4*) and type‐4 (higher at 18°C, e.g., *LesaXTH24*) temperature‐specific patterns were retained, and for some type‐4 DEGs (e.g., *LesaXTH19* and *LesaXTH33*) roughly equal transcript abundances were obtained for 11°C and 18°C (cold temperatures), but not for the optimal temperature (27°C). Simple thermal‐time compliant patterns would deliver equal transcript abundances in the RT‐qPCR verification at 270°C·h, but this was not the case. Except for *LesaEXPA2*, which is not expressed in the RAD, none of the identified CAP CWRP DEG candidates exhibited fully thermal‐time compliant gene expression patterns across the entire sub‐optimal, optimal and supra‐optimal temperature range. *LesaEXPA2* seem to exhibit such a pattern, but this was not associated with endosperm weakening at supra‐optimal temperatures. The decrease in GR values in the supra‐optimal temperature range is known to be associated with changes in seed water relations and thermoinhibition at *T*_c_ (Alvarado and Bradford 2002; Bradford and Bello 2022; Rowse and Finch‐Savage 2003; Watt and Bloomberg 2012; Watt et al. 2011). It was observed that while the base water potential *Ψ*_b_ is constant at sub‐optimal temperatures, at supra‐optimal temperatures (above *T*_o_), it increases linearly with temperature. Rowse and Finch‐Savage (2003) proposed that thermal‐time continues to accumulate above *T*_o_, but is offset by the concurrent increase in base water potential and thermoinhibition occurs when it reaches 0 MPa at *T*_c_. Water relations in the embryo growth zone of the RAD therefore differ between sub‐optimal temperatures and supra‐optimal temperatures. Possible thermal‐time compliant gene expression patterns in the RAD were only observed for some genes encoding XG‐targeting enzmes (e.g., *LesaXXT1/2*, *LesaβGAL8*, *LesaCSLC4* and *LesaXTH16*), but not for the majority of CWRP genes (Figures S5–S9). If the cumulative transcript abundances of each individual CWRP group were used as proxy for expansin or enzymatic activity, also their patterns did not reveal (except for possibly FUTs, βGALs, PLLs, and PMEIs in the CAP) any obvious group with perfectly matched expression on a HU basis in the CAP (endosperm weakening) or in the RAD (increase in embryo growth potential due to cell‐wall loosening in the growth zone) between the temperature and the time course transcriptomes (Figure S10). Taken together, simple thermal‐time compliant gene expression patterns across the entire sub‐optimal temperature region were not observed for the majority of identified *L. sativum* CWRP DEGs. Our results therefore support the view that distinct molecular and biomechanical mechanisms are underpinning the apparently linear thermal‐time responses (germination rates) during CAP weakening.

## Conflicts of Interest

The authors declare no conflicts of interest.

## Supporting information

SupportingInfoSteinbrecher2025‐PCE.

**SI Dataset S1.** Transcriptome abundances in the *Lepidium sativum* FR14 seed compartments of the temperature arrays.

**SI Dataset S2.** *Lepidium sativum* DEGs: 11ºC versus 18ºC.

**SI Dataset S3.** *Lepidium sativum* DEGs: 11ºC versus 27ºC.

**SI Dataset S4.** *Lepidium sativum* DEGs: 11ºC versus 32ºC.

**SI Dataset S5.** *Lepidium sativum* DEGs: 18ºC versus 27ºC.

**SI Dataset S6.** *Lepidium sativum* DEGs: 18ºC versus 32ºC.

**SI Dataset S7.** *Lepidium sativum* DEGs: 27ºC versus 32ºC.

**SI Dataset S8.** *Lepidium sativum* CAP DEGs in the time course arrays.

**SI Dataset S9.** *Lepidium sativum* CAP UP‐regulated DEGs in the time course arrays.

**SI Dataset S10.** *Lepidium sativum* CAP DEGs common to the temperature and time course arrays.

**SI Dataset S11.** *Lepidium sativum* DEGs unique to the temperature arrays.

**SI Dataset S12.** *Lepidium sativum* CAP CWRP DEGs common to the temperature and time course arrays.

**SI Dataset S13.** *Lepidium sativum* CAP CWRP DEGs unique to the temperature arrays.

## Data Availability

The data that support the findings of this study are openly available in ArrayExpress Database at http://www.ebi.ac.uk/arrayexpress, reference number E‐MTAB‐1811. Microarray data are available in the ArrayExpress database (www.ebi.ac.uk/arrayexpress) under accession number E‐MTAB‐1811.
