# Supplementary material for: Distinct Molecular Biomechanical Mechanisms Inhibit Endosperm Cell‐Wall Weakening and Seed Germination at Cold and Warm Nonoptimal Temperatures
Source: Plant Cell Environ. 2025 Aug 5;48(11):8047–67. doi: 10.1111/pce.70103 (PMC12502036; doi:10.1111/pce.70103)
Supplement: Supplementary file 1 — SupportingInfoSteinbrecher2025‐PCE. [file PCE-48-8047-s001.pdf]

***Plant, Cell & Environment* 2025 – Supporting Information (SI)**

Distinct molecular biomechanical mechanisms inhibit endosperm cell-wall weakening and seed germination at cold and warm non-optimal temperatures

Tina Steinbrecher, Antje Voegele, Michael Ignatz, Karin Weitbrecht, Safina Khan, Kai Graeber, James E. Hourston and Gerhard Leubner-Metzger

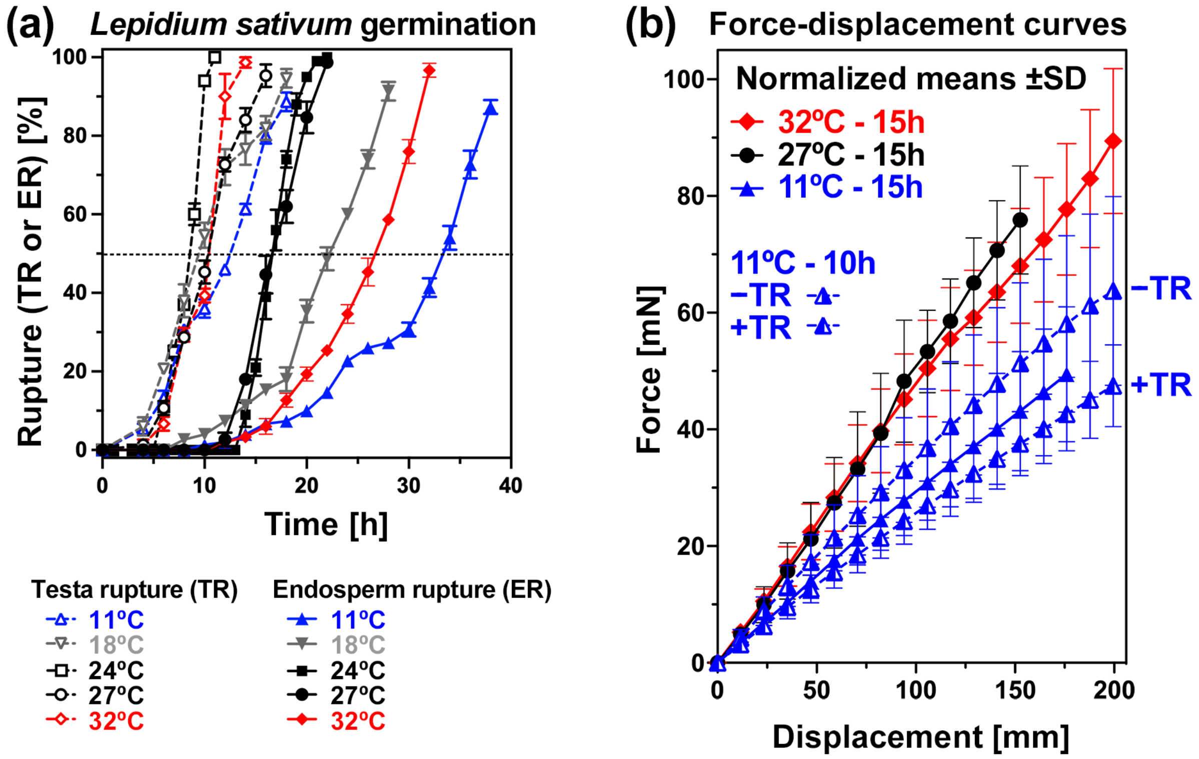

Fig. S1. The effect of imbibition temperature on germination and biomechanical properties of the non-dormant *Lepidium sativum* seed population.

(a) Time course of testa rupture (TR) and endosperm rupture (ER) of seeds incubated at 11°C, 18°C, 24ºC, 27°C and 32°C in continuous white light. Mean values ± SE of 3 x 50 seeds are shown. Note that the time course of TR did not appreciably differ between the five temperatures until 50% TR. For the transcriptome sampling at 10 h all seeds had unruptured CAPs and ~50% of the seeds were without (-TR) and with (+TR) testa rupture.

(b) Linear regions of the force-displacement curves, normalized means ±SD of >20 curves of CAPs dissected at 15 h at the temperatures indicated. The stiffness values (Fig. 2c) are derived from the slopes of the curves. Note that at the cold temperature TR is associated with a decreased slope (decreased CAP stiffness).

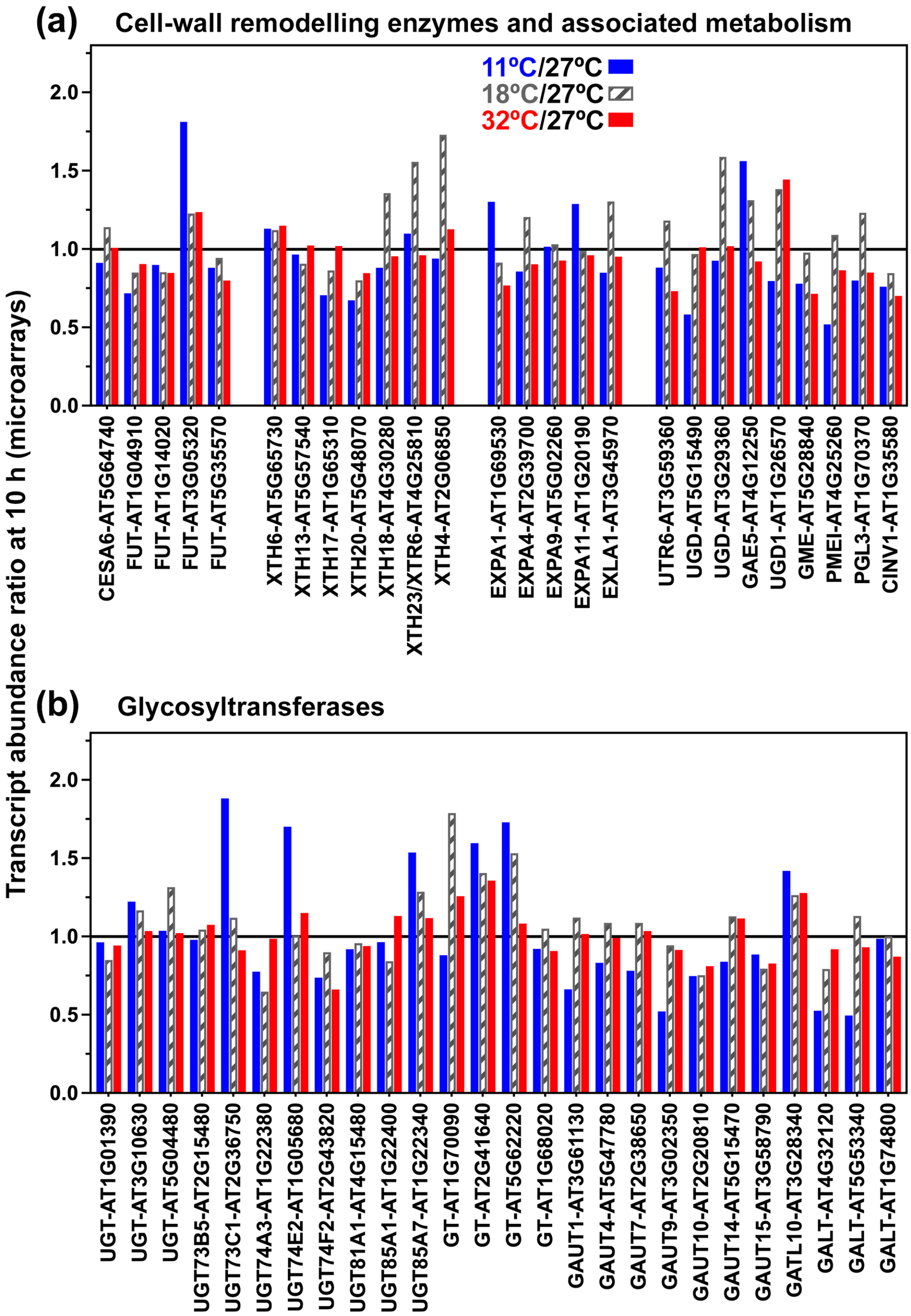

Fig. S2. Expression ratios in the *Lepidium sativum* temperature transcriptomes at 10 h compared to the optimal temperature (27ºC) of CAP CWRP genes identified as DEGs (transcript abunance ratios ≥2 or ≤0.5) unique to the time course transcriptomes. Of the 58 CAP CWRP DEGs unique to the time course transcriptomes, 53 were also present in the temperature microarrays with expression ratios transcript abundance ratios between <2 and >0.5 (in many cases ~1). AGI codes of putative *Arabidopsis thaliana* orthologs are shown as part of the *L. sativum* transcript names.

(a) Genes encoding cellulose synthase (*CESA*), α-fucosyltransferase (*FUT*), expansins (*EXP*), UPD-galactose transporter (UTR), UDP-glucose dehydrogenase (*UGD*), UDP-D-glucuronate 4-epimerase (*GAE*), GDP-D-mannose 3',5'-epimerase (*GME*), pectin methylesterase inhibitor (*PMEI*) and an invertase (*CINV1*).

(b) Genes encoding UDP-glycosyltransferases (*UGT*), glycosyltransferases (*GT*), galacturonosyl-transferases (*GAUT*, *GalT*), galacturonosyltransferase-like (*GATL*) and hydroxyproline O-galactosyltransferases (*GALT*).

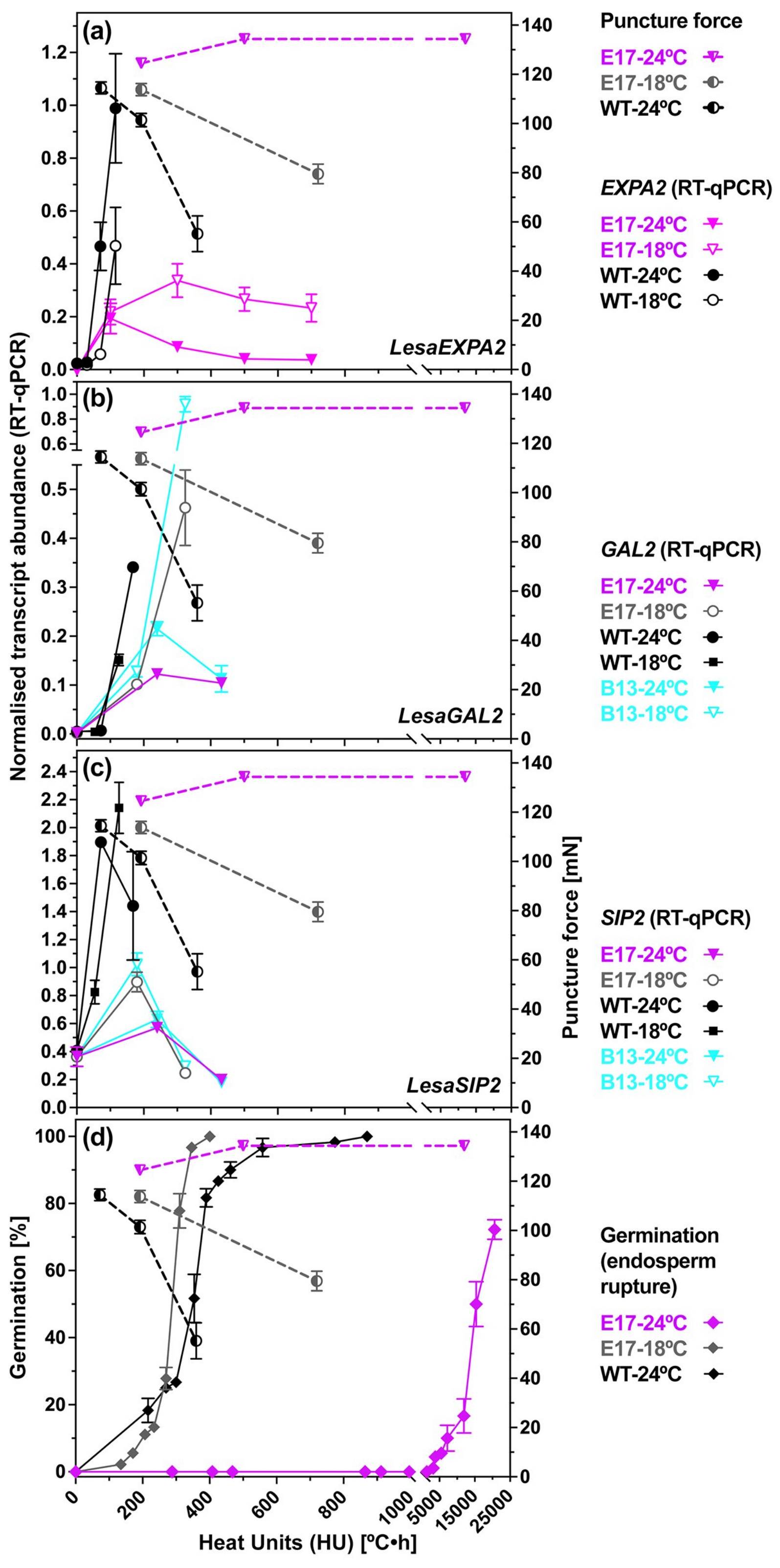

Fig. S3. Gene expression (RT-qPCR) and biomechanical (CAP puncture force) analysis in *Lepidium sativum* FR14 (wild type, WT) and in DOG1-overexpressing transgenic *L. sativum* E17 and B13 seeds imbibed at 18ºC and 24ºC presented along a heat unit (HU in ºC•h above T_b = 0ºC) scale x-axis. (a) *LesaEXPA2* transcript abundances. (b) *LesaGAL2* transcript abundances. (c) *LesaSIP2* transcript abundances. (d) Germination kinetics and CAP puncture force values (Graeber *et al.*, 2014) presented along a HU scale x-axis. Mean ± SEM values are shown (N = 4 for RT-qPCR).

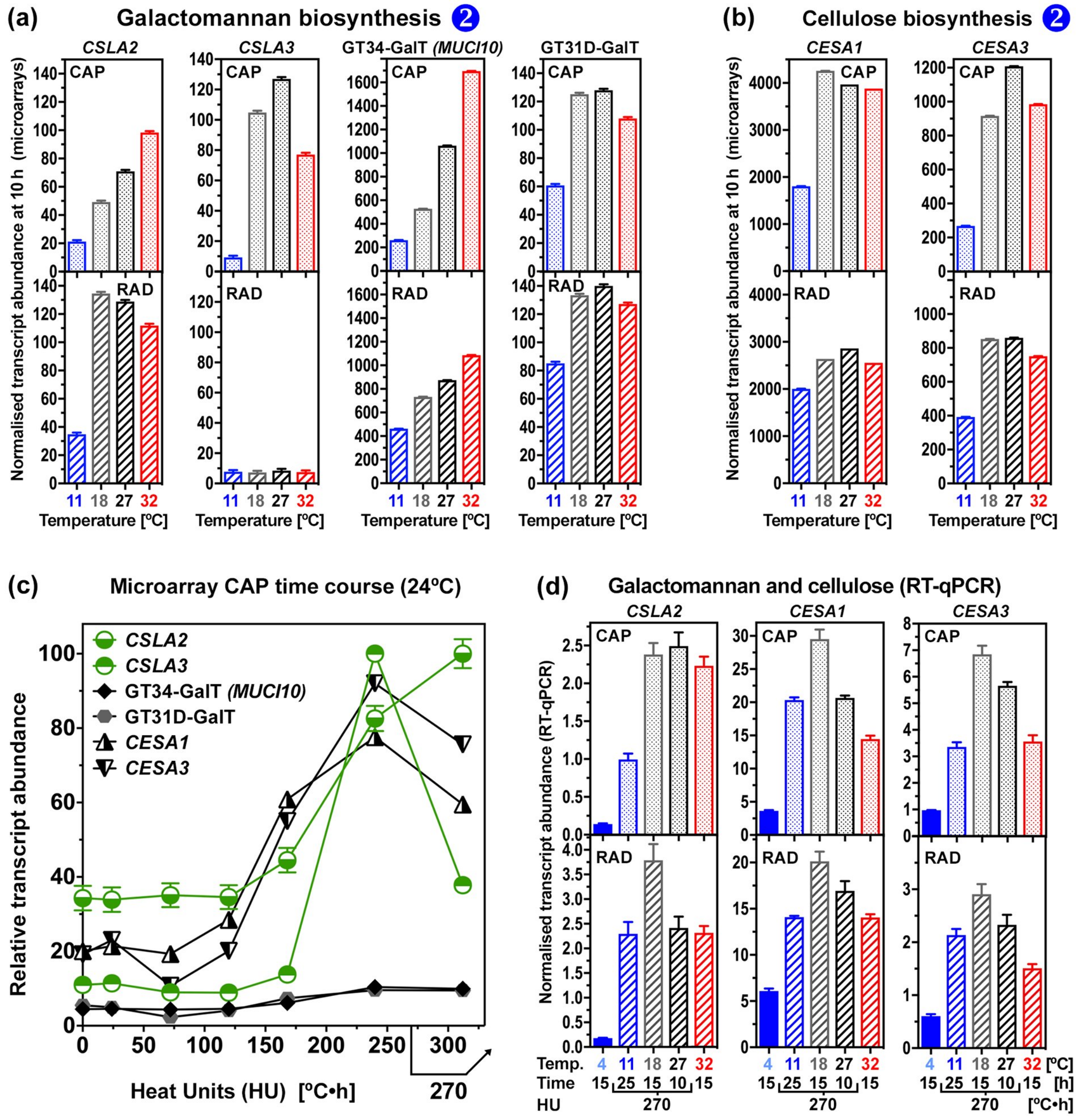

Fig. S4. Temperature regulation of galactomannan and cellulose biosynthesis genes during *Lepidium sativum* seed germination.

(a) Transcript abundances of galactomannan biosynthesis genes in the CAP (micropylar endosperm) and RAD (radicle and lower hypocotyl) in the temperature transcriptomes (microarrays) at 10 h at the temperatures indicated. Note that these genes were identified to be type-2 (down at 11ºC) CAP weakening DEGs. *Abbreviations:* cellulose synthase (CESA), cellulose synthase like-A (CSLA), galacturonosyltransferase (GalT). Mean ± SEM values (N = 4).

(b) Transcript abundances of cellulose biosynthesis genes in the CAP and RAD in the temperature transcriptomes. Note that these genes were identified to be type-2 (down at 11ºC) CAP weakening DEGs. Mean ± SEM values (N = 4).

(c) CAP transcript abundances of galactomannan and cellulose biosynthesis CAP DEGs in the time course (microarrays at 24ºC) transcriptomes presented along a heat unit (HU in ºC•h above T_b = 0ºC) scale as x-axis. The 270 ºC•h HU value is indicated as it was used to conduct RT-qPCR analysis in an independent experiment (*see panel d*).

(d) RT-qPCR analysis of *LesaGAL2* at 15 h (4ºC, 18ºC, 32ºC) and at an HU value of 270 ºC•h HU value generated by different time-temperature combinations as indicated. The transcript abundances differed in the CAP at 270 ºC•h in the sub-optimal temperature range indicating that these DEGs are not expressed in a thermal time compliant manner. Mean ± SEM values (N = 4).

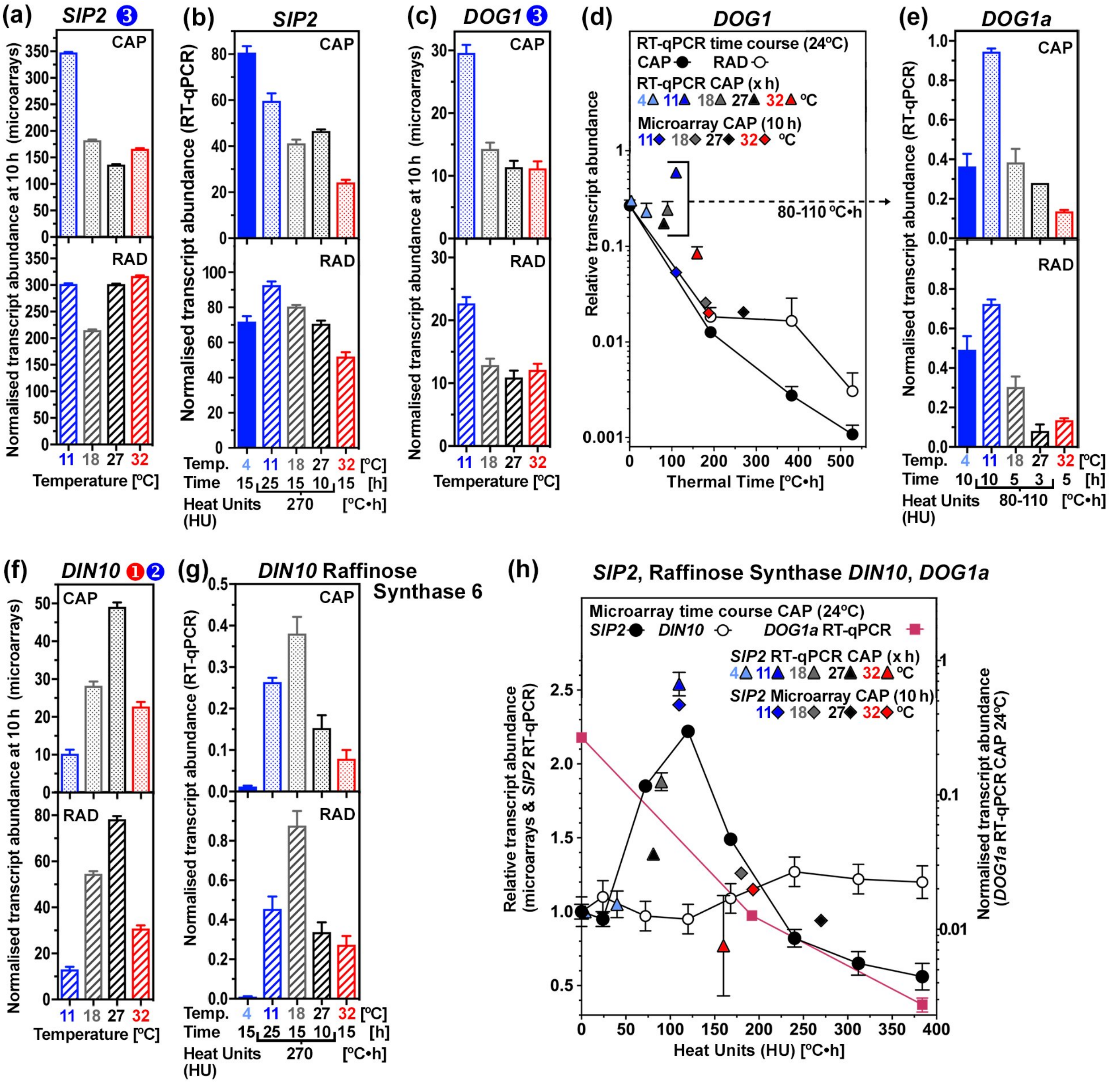

Fig. S5. Temperature regulation of raffinose cycle genes and the *DOG1* gene during *Lepidium sativum* seed germination.

(a) Transcript abundances of the α-galactosidase *LesaSIP2* (*SEED IMBIBITION 2*) type-3 (up at 11ºC, stiffening/elasticity gene) CAP DEG in the CAP (micropylar endosperm) and RAD (radicle and lower hypocotyl) in the temperature transcriptomes (microarrays) at 10 h at the temperatures indicated. Mean ± SEM values (N = 4).

(b) RT-qPCR analysis of *LesaSIP2* transcript abundances at 15 h (4ºC, 18ºC, 32ºC) and at an HU value of 270 ºC•h HU value generated by different time-temperature combinations as indicated. Mean ± SEM values (N = 4). The *LesaSIP2* transcript abundances differed in the CAP at 270 ºC•h in the sub-optimal temperature range indicating that *LesaSIP2* is not expressed in a thermal time compliant manner.

(c) Transcript abundances of the *LesaDOG1* (*DELAY OF GERMINATION 1*) (DOG1) type-3 (up at 11ºC, stiffening/elasticity gene) CAP DEG in the CAP and RAD in the temperature transcriptomes. Mean ± SEM values (N = 4).

(d) CAP and RAD transcript abundances of *LesaDOG1* in an RT-PCR generated time course (24ºC), temperature microarray and RT-qPCR at different temperature-time combinations presented along a heat unit (HU in ºC•h above T_b = 0ºC) scale as x-axis. Mean ± SEM values (N = 4) are presented. The 80-110 ºC•h HU value is indicated as it was used to conduct RT-qPCR analysis in an independent experiment (*see panel e*).

(e) RT-qPCR analysis of *LesaDOG1a* transcript abundances at different temperature-time combinations including HU values of 80-110 ºC•h as indicated. Mean ± SEM values (N = 4) are presented. The transcript abundances differed in the CAP at 80-110 ºC•h in the sub-optimal temperature range indicating that *LesaDOG1a* is not expressed in a thermal time compliant manner.

(f) Transcript abundances of the raffinose synthase 6 (*DIN10*, *DARK INDUCIBLE 10*) type-1 (down at 32ºC) and type-2 (down at 11ºC) CAP DEG in the CAP and RAD in the temperature transcriptomes. Mean ± SEM values (N = 4).

(g) RT-qPCR analysis of *DIN10* raffinose synthase transcript abundances at 15 h (4ºC, 18ºC, 32ºC) and at an HU value of 270 ºC•h HU value generated by different time-temperature combinations as indicated. Mean ± SEM values (N = 4). The transcript abundances differed in the CAP at 270 ºC•h in the sub-optimal temperature range indicating that the raffinose synthase is not expressed in a thermal time compliant manner.

(h) CAP transcript abundances of *LesaSIP2*, *LesaDIN10* and *LesaDOG1a* in the microarray time course (24ºC) and by RT-qPCR at different temperature-time combinations presented along a HU scale x-axis. Mean ± SEM values (N = 4) are presented.

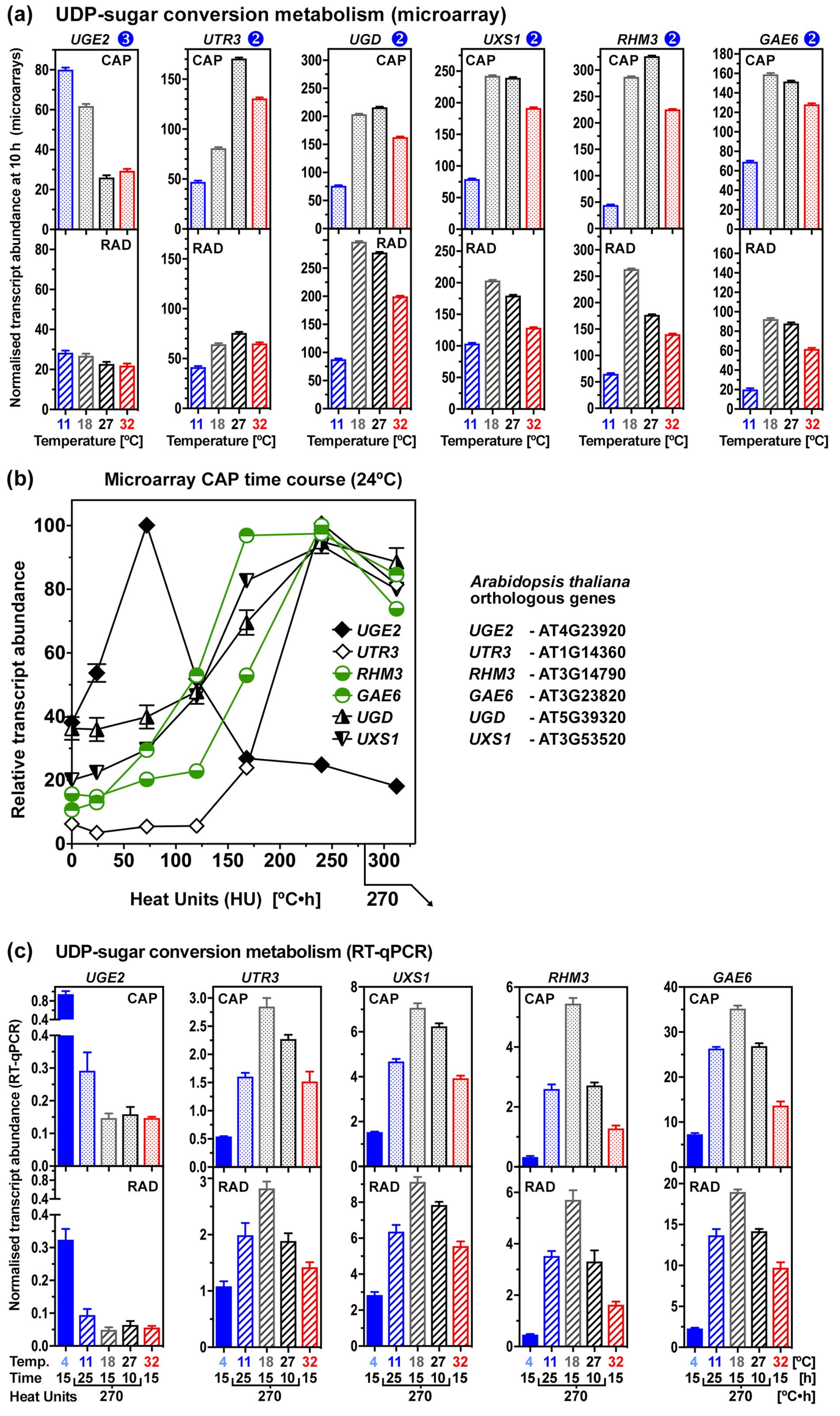

Fig. S6. Temperature regulation of UDP-sugar metabolism genes during *Lepidium sativum* seed germination.

(a) Transcript abundances of UDP-sugar metabolism genes in the CAP (micropylar endosperm) and RAD (radicle and lower hypocotyl) in the temperature transcriptomes (microarrays) at 10 h at the temperatures indicated. Mean ± SEM values (N = 4). Note that these genes were identified to be type-2 (down at 11ºC) and type-3 (up at 11ºC, stiffening/elasticity gene) CAP weakening DEGs, as indicated. *Abbreviations:* UDP-glucose dehydrogenase (UGD), UDP-D-glucuronate 4-epimerase (GAE), UDP-D-glucose 4-epimerase (UGE), UDP-glucuronic acid decarboxylase (UXS; enzyme produces UDP-xylose), UPD-galactose transporter (UTR), rhamnose biosynthesis (RHM).

(b) CAP transcript abundances of UDP-sugar metabolism CAP DEGs in the time course (microarrays at 24ºC) transcriptomes presented along a heat unit (HU in ºC•h above T_b = 0ºC) scale x-axis. Mean ± SEM values (N = 4) are presented. The 270 ºC•h HU value is indicated as it was used to conduct RT-qPCR analysis in an independent experiment (*see panel c*).

(c) RT-qPCR analysis of UDP-sugar metabolism gene transcript abundances at 15 h (4ºC, 18ºC, 32ºC) and at an HU value of 270 ºC•h HU value generated by different time-temperature combinations as indicated. Mean ± SEM values (N = 4). The transcript abundances differed in the CAP at 270 ºC•h in the sub-optimal temperature range indicating that these DEGs are not expressed in a thermal time compliant manner.

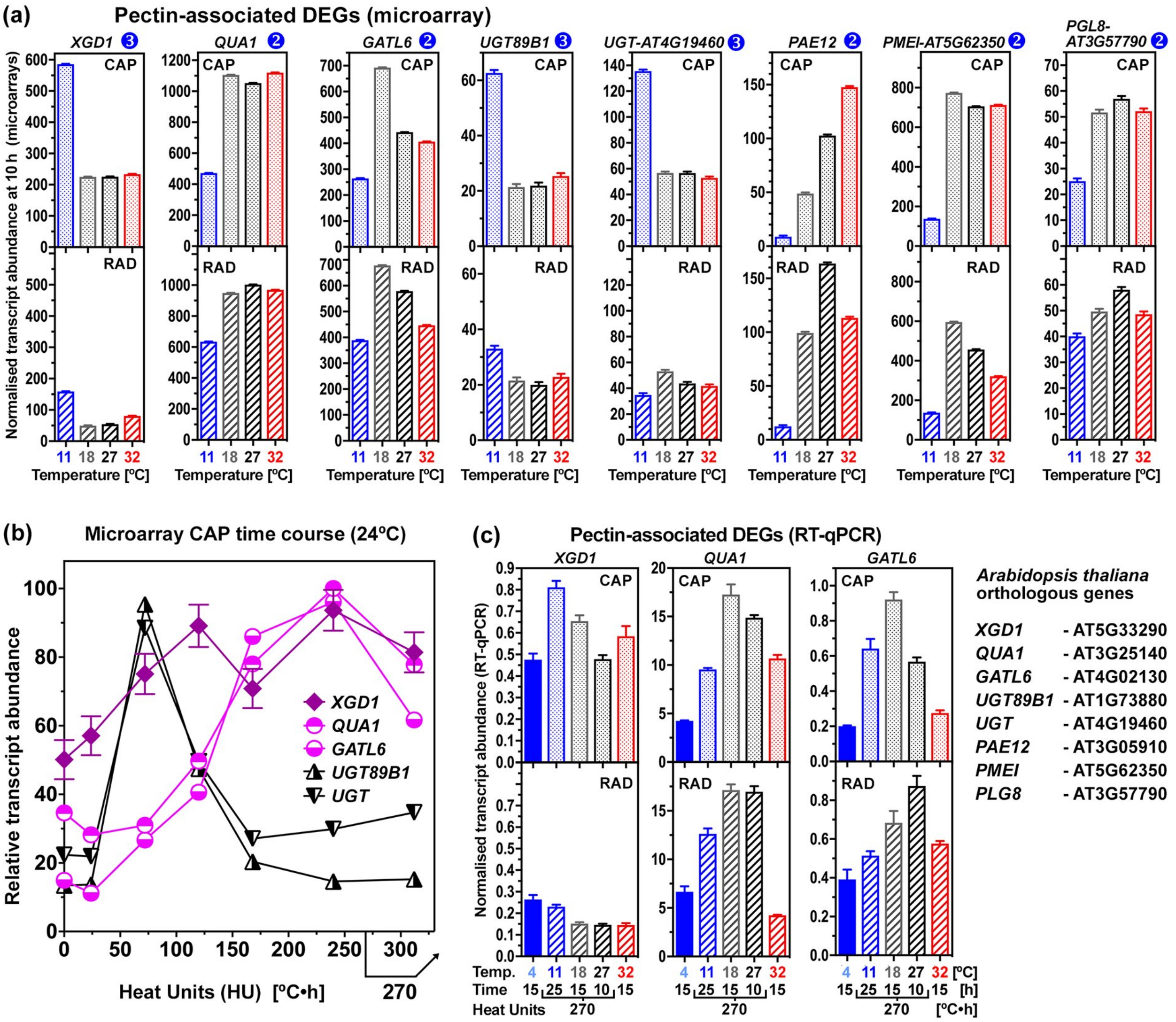

Fig. S7. Temperature regulation of pectin-related genes during *Lepidium sativum* seed germination.

(a) Transcript abundances of pectin-related genes in the CAP (micropylar endosperm) and RAD (radicle and lower hypocotyl) in the temperature transcriptomes (microarrays) at 10 h at the temperatures indicated. Mean ± SEM values (N = 4). Note that these genes were identified to be type-2 (down at 11ºC) and type-3 (up at 11ºC, stiffening/elasticity gene) CAP weakening DEGs, as indicated. *Abbreviations:* galacturonosyltransferase QUA1 (QUASIMODO 1), xylogalacturonan (XGA) xylosyltransferase XGD1 (XGA DEFICIENT 1), galacturonosyl-transferase-like (GATL), UDP-glycosyltransferase (UGT), pectinacetylesterase (PAE), pectin methylesterase inhibitor (PMEI), pectin lyase-like/polygalacturonase-like (PGL).

(b) CAP transcript abundances of pectin-related CAP DEGs in the time course (microarrays at 24ºC) transcriptomes presented along a heat unit (HU in ºC•h above T_b = 0ºC) scale x-axis. Mean ± SEM values (N = 4) are presented. The 270 ºC•h HU value is indicated as it was used to conduct RT-qPCR analysis in an independent experiment (*see panel c*).

(c) RT-qPCR analysis of pectin-related gene transcript abundances at 15 h (4ºC, 18ºC, 32ºC) and at an HU value of 270 ºC•h HU value generated by different time-temperature combinations as indicated. Mean ± SEM values (N = 4). The transcript abundances differed in the CAP at 270 ºC•h in the sub-optimal temperature range indicating that these DEGs are not expressed in a thermal time compliant manner.

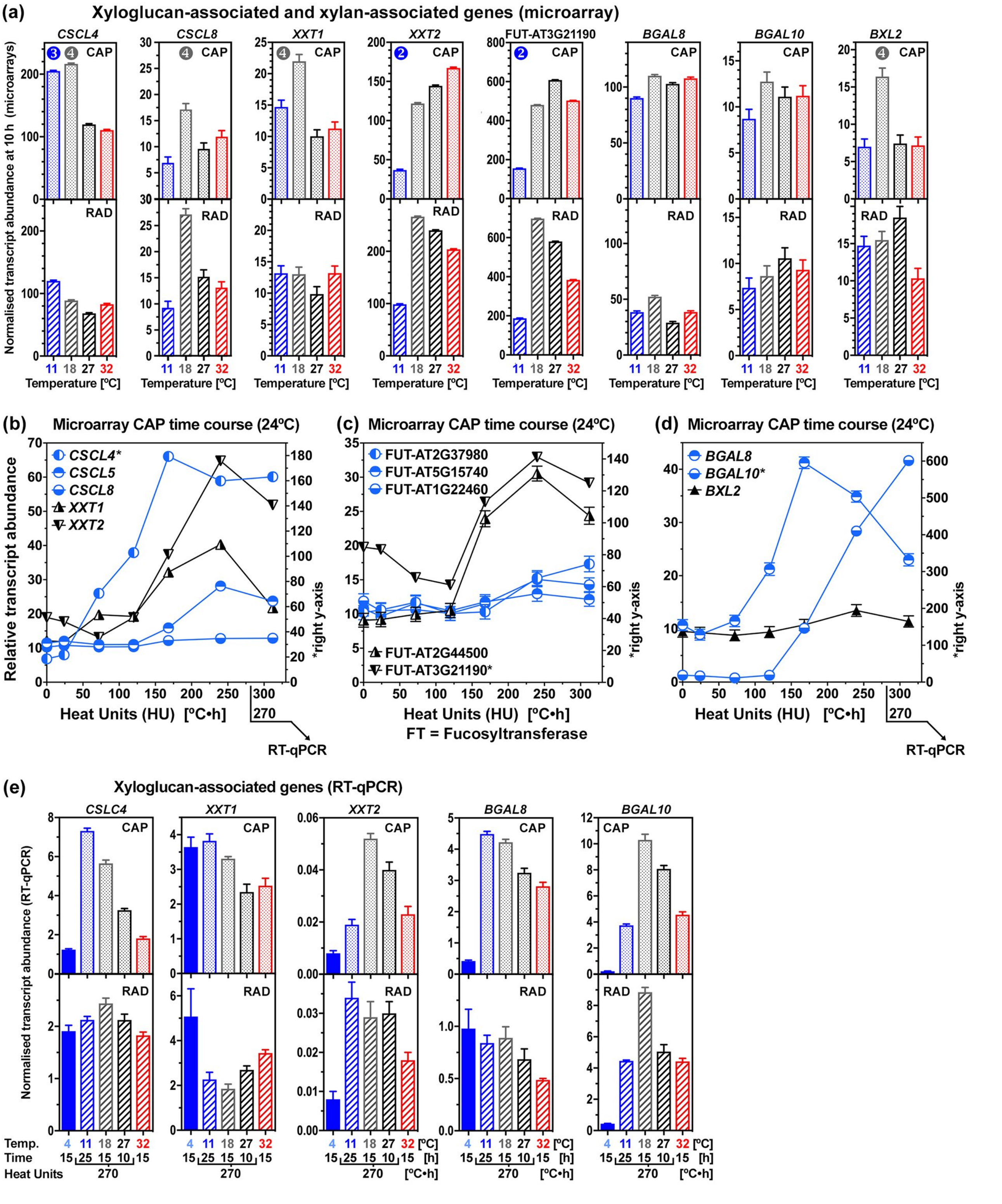

Fig. S8. Temperature regulation of xyloglucan (XG) and xylan (*BXL2*) associated genes during *Lepidium sativum* seed germination.

(a) Transcript abundances of XG-related genes and *LesaBXL2* in the CAP (micropylar endosperm) and RAD (radicle and lower hypocotyl) in the temperature transcriptomes (microarrays) at 10 h at the temperatures indicated. Mean ± SEM values (N = 4). Note that most of these genes were identified to be type-2 (down at 11ºC), type-3 (up at 11ºC, stiffening/elasticity gene) and type-4 (up at 18ºC) CAP weakening DEGs, as indicated. *Abbreviations:* XG xylosyltransferase (XXT), cellulose synthase like-C (CSLC), α-fucosyltransferase (FUT), β-galactosidase (BGAL), β-xylosidase (BXL).

(b-d) CAP transcript abundances of XG-related genes and *LesaBXL2* in the time course (microarrays at 24ºC) transcriptomes presented along a heat unit (HU in ºC•h above T_b = 0ºC) scale x-axis. Mean

± SEM values (N = 4) are presented. The 270 ºC•h HU value is indicated as it was used to conduct RT-qPCR analysis in an independent experiment (*see panel c*).

(e) RT-qPCR analysis of XG-related gene transcript abundances at 15 h (4ºC, 18ºC, 32ºC) and at an HU value of 270 ºC•h HU value generated by different time-temperature combinations as indicated. Mean ± SEM values (N = 4). The transcript abundances differed in the CAP at 270 ºC•h in the sub-optimal temperature range indicating that these genes are not expressed in a thermal time compliant manner.

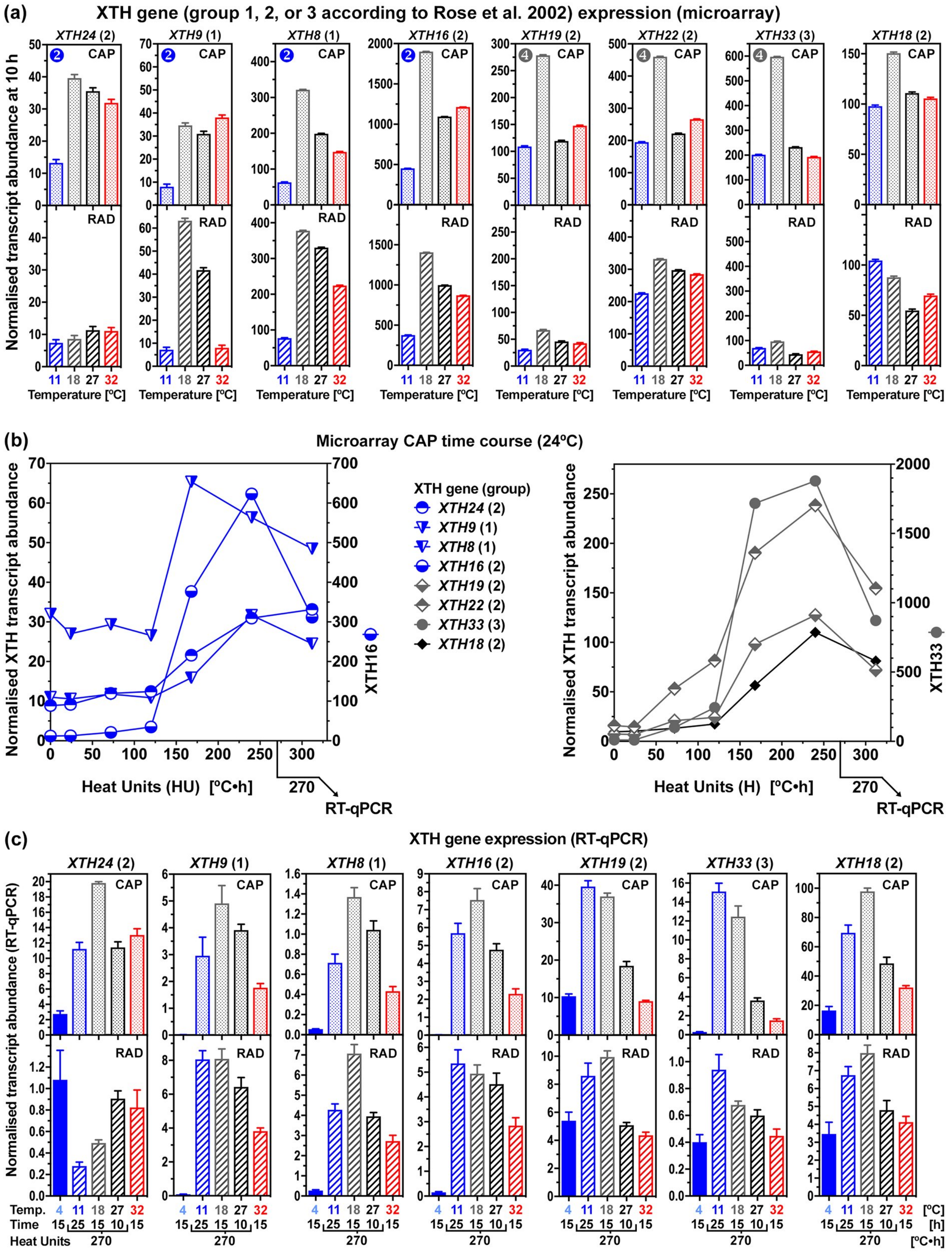

Fig. S9. Temperature regulation of xyloglucan (XG) endotransglycolase/hydrolase (XTH) genes during *Lepidium sativum* seed germination.

(a) Transcript abundances of XTH DEGs in the CAP (micropylar endosperm) and RAD (radicle and lower hypocotyl) in the temperature transcriptomes (microarrays) at 10 h at the temperatures indicated. Mean ± SEM values (N = 4). Note that most of these genes were identified to be type-2 (down at 11ºC) or type-4 (up at 18ºC) CAP weakening DEGs, as indicated. XTH group numbers (Rose *et al.*, 2002) are indicated in brackets.

(b) CAP transcript abundances of XTH CAP DEGs in the time course (microarrays at 24ºC) transcriptomes presented along a heat unit (HU in ºC•h above T_b = 0ºC) scale x-axis. Mean ± SEM values (N = 4) are presented. The 270 ºC•h HU value is indicated as it was used to conduct RT-qPCR analysis in an independent experiment (*see panel c*).

(c) RT-qPCR analysis of XTH CAP DEG transcript abundances at 15 h (4ºC, 18ºC, 32ºC) and at an HU value of 270 ºC•h HU value generated by different time-temperature combinations as indicated. Mean ± SEM values (N = 4). The transcript abundances differed in the CAP at 270 ºC•h in the sub-optimal temperature range indicating that these XTH DEGs are not expressed in a thermal time compliant manner.

Rose JK, Braam J, Fry SC, Nishitani K. 2002. The XTH family of enzymes involved in xyloglucan endotransglucosylation and endohydrolysis: current perspectives and a new unifying nomenclature. *Plant & Cell Physiology* **43**(12): 1421-1435.

Fig. S10:

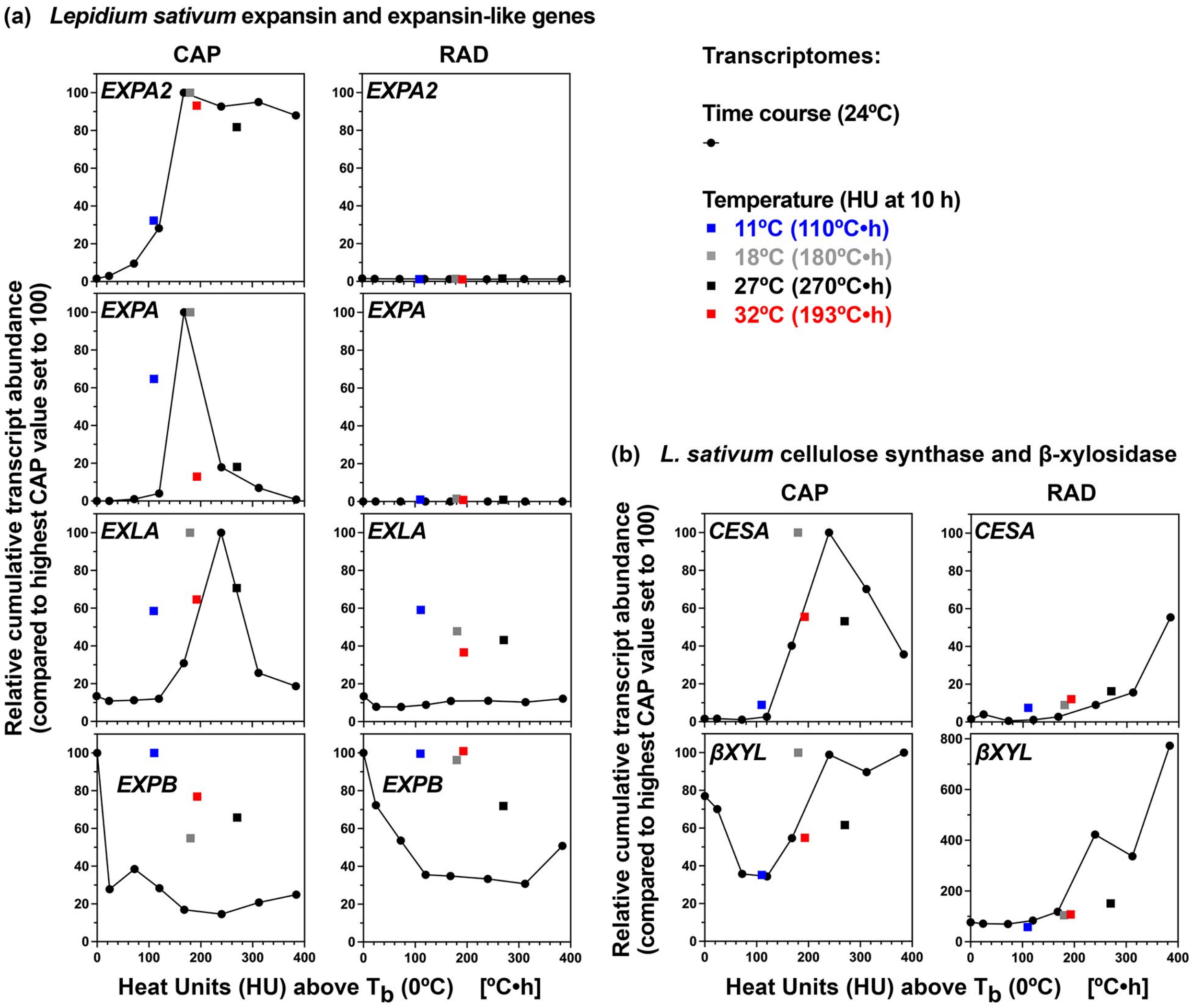
(a) Lepidium sativum expansin and expansin-like genes
CAP
RAD
EXPA2
EXPA
EXLA
EXPB
Relative cumulative transcript abundance
(compared to highest CAP value set to 100)
Heat Units (HU) above Tb (0ºC) [ºC•h]
Transcriptomes:
Time course (24ºC)
Temperature (HU at 10 h)
11ºC (110ºC•h)
18ºC (180ºC•h)
27ºC (270ºC•h)
32ºC (193ºC•h)
(b) L. sativum cellulose synthase and β-xylosidase
CAP
RAD
CESA
βXYL
Relative cumulative transcript abundance
(compared to highest CAP value set to 100)
Heat Units (HU) above Tb (0ºC) [ºC•h]

Fig. S10:

(c) Xyloglucan (XG) example structure and expression of *L. sativum* genes encoding XG-modifying enzymes

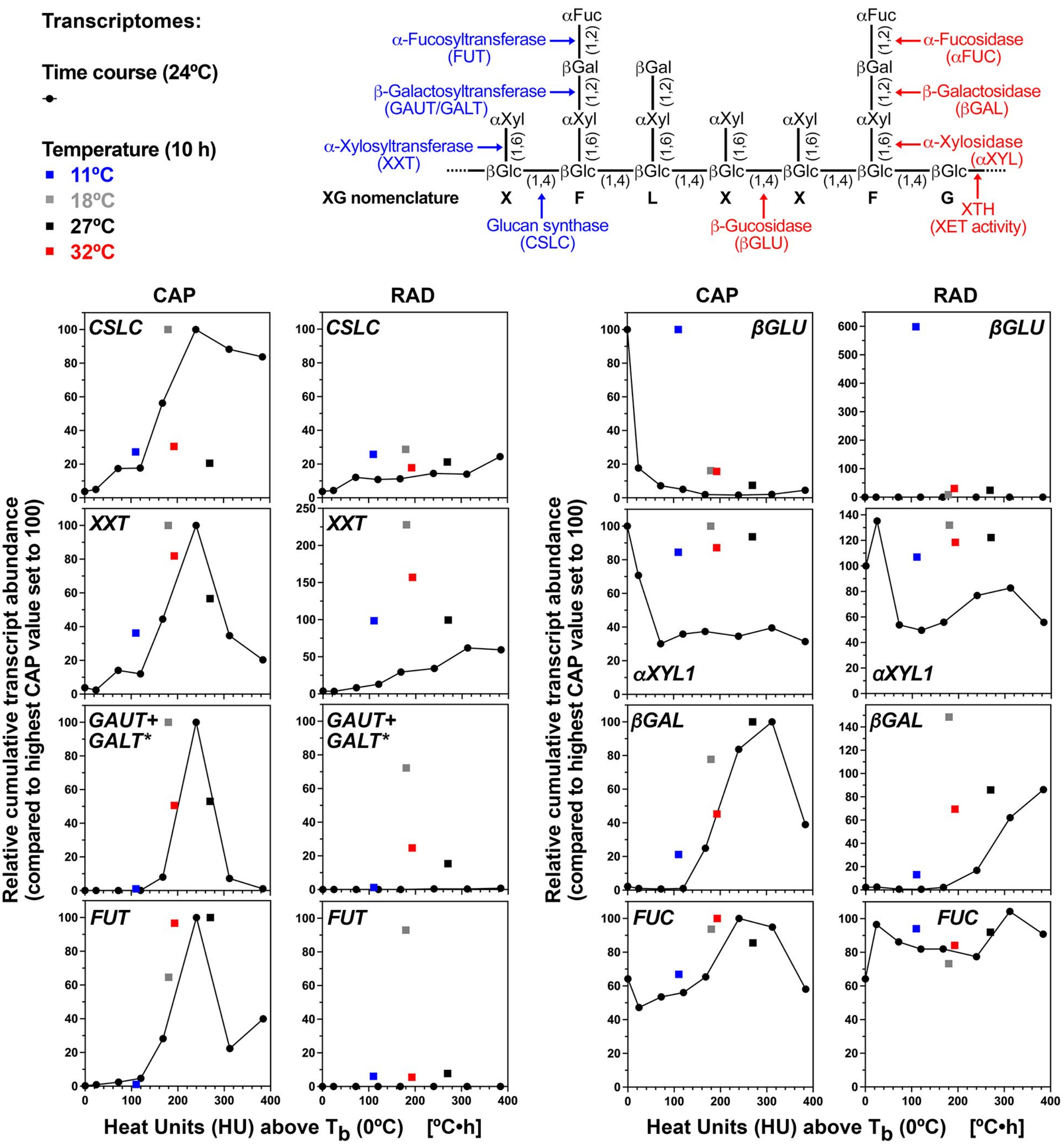

* Galacturonosyltransferases (GAUT+GATL) have XG and/or pectins as target

Fig. S10:

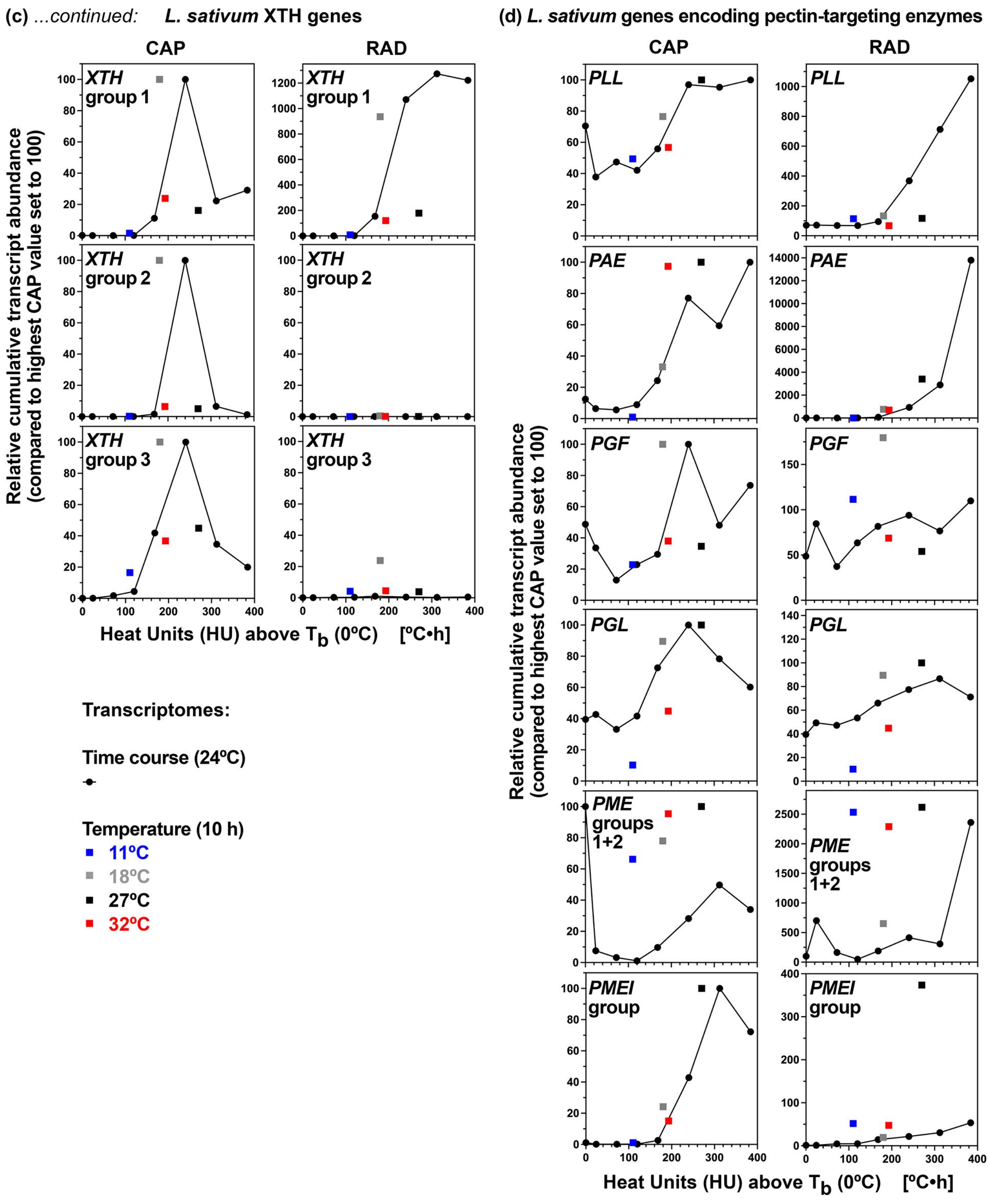

Fig. S10. Cumulative transcript abundance from the time course (24ºC) and temperature (10 h) transcriptomes of all identified CWRP gene groups in the (micropylar endosperm) and RAD (radicle and lower hypocotyl) during *Lepidium sativum* seed germination presented along a heat unit (HU in ºC•h above T_b = 0ºC) scale x-axis. The accumulated thermal-time above Tb at 10 h was 110ºC•h (at 10ºC), 180ºC•h (at 18ºC), and 270ºC•h (at 27ºC) in the sub-optimal to optimal temperature range. For the accumulated thermal-time above Tb at 10 h at 32ºC we used 193ºC•h (*see main text of the*

manuscript for calculation details). AGI codes of putative *Arabidopsis thaliana* orthologs are listed for the *L. sativum* transcripts used to calculate the cumulative transcript abundances.

(a) Expression of α-expansin (EXPA), β-expansin (EXPB) and expansin-like (EXLA) genes.

EXPA: AT1G69530, AT5G05290 (*EXPA2*), AT2G37640, AT2G39700, AT2G28950, AT1G12560, AT2G40610, AT5G02260, AT1G26770, AT1G20190, AT3G15370, AT3G03220, AT5G56320, AT2G03090, AT3G55500, AT4G01630, AT1G62980, AT4G38210, AT5G39260. EXPB: AT2G20750, AT1G65680, AT4G28250, AT3G60570. EXLA: AT3G45970, AT4G38400, AT3G45960.

(b) Expression of cellulose synthase (CESA) and β-xylosidase (BXL, βXYL) genes.

CESA: AT5G64740, AT2G21770, AT5G09870, AT4G32410, AT5G05170, AT2G25540, AT5G44030, AT5G17420, AT4G18780. βXYL: AT5G64570, AT1G02640, AT5G49360, AT5G09730, AT5G10560, AT1G68560.

(c) Xyloglucan (XG) example structure and expression of XG-biosynthesis and XG-modifying enzyme genes. XG xylosyltransferase (XXT), cellulose synthase like-C (CSLC), α-fucosyltransferase (FUT), β-glucanase (βGLU), α-xylosidase (αXYL1), β-galactosidase (βGAL), α-fucosidase (FUC), XG endotransglycolase/hydrolase (XTH), galacturonosyl-transferase (GAUT), galacturonosyltransferase-like (GATL).

XXT: AT3G62720, AT4G02500, AT5G07720, AT1G18690, AT1G74380. CSLC: AT4G07960, AT3G28180, AT4G31590, AT2G24630. FUT: AT1G04910, AT1G14020, AT1G14080, AT1G22460, AT1G29200, AT1G35510, AT1G49710, AT1G53770, AT1G62330, AT1G71990, AT2G01480, AT2G03210, AT2G18760, AT2G44500, AT3G03810, AT3G05320, AT3G21190, AT3G26370, AT3G54100, AT4G16650, AT4G17430, AT5G15740, AT5G35570, AT5G62550, AT5G65470. βGLU: AT1G02850, AT1G75940, AT2G44450, AT2G44460, AT2G44490, AT3G03640, AT3G09260, AT3G18080, AT3G21370, AT3G24180, AT4G22100, AT4G27820, AT5G28510, AT5G36890, AT5G54570. αXYL1: AT1G68560. βGAL: AT3G13750, AT5G63810, AT4G38590, AT1G77410, AT3G52840, AT4G36360, AT5G56870, AT1G45130, AT2G28470, AT2G32810. FUC: AT1G67830, AT2G28100, AT4G34260. GAUT/GATL: AT1G53290, AT1G74800, AT1G22015, AT5G62620, AT1G27120, AT3G06440, AT5G53340, AT1G32930, AT2G38650, AT1G18580, AT2G32580, AT4G38270, AT5G15470, AT1G06780, AT3G02350, AT3G58790, AT3G01040, AT2G20810, AT3G25140, AT3G61130, AT5G47780. XTH-group1: AT4G13080, AT2G06850, AT5G13870, AT5G65730, AT1G11545, AT4G03210, AT2G14620. XTH-group2: AT5G57540, AT4G14130, AT3G23730, AT1G65310, AT4G30280, AT4G30290, AT5G48070, AT5G57560, AT4G25810, AT4G30270, AT5G57550. XTH-group3: AT2G01850, AT1G14720, AT1G32170, AT3G44990, AT2G36870, AT1G10550.

(d) Expression of pectin-related genes. Pectin lyase (PLL), pectinacetylesterase (PAE), pectin lyase-like/polygalacturonase-like (PGL), polygalacturonase (PGF), pectin methylesterase (PME), pectin methylesterase inhibitor (PMEI).

PLL: AT3G01270, AT4G24780, AT1G67750, AT3G27400, AT5G04310, AT3G54920. PAE: AT5G45280, AT2G46930, AT1G57590, AT4G19410, AT3G62060, AT3G05910. PGL: AT1G56710, AT1G60390, AT1G70370, AT1G23760, AT5G14650. PGF: AT4G33440, AT5G41870, AT1G19170, AT2G23900, AT3G57790, AT3G42950, AT3G61490, AT3G06770, AT3G48950. PME (groups 1+2): AT1G44980, AT2G19150, AT3G29090, AT5G55590, AT1G02810, AT1G11590, AT1G53830, AT1G53840, AT2G26440, AT2G26450, AT2G43050, AT2G47550, AT3G10710, AT3G14300, AT3G14310, AT3G43270, AT3G47400, AT3G49220, AT3G62170, AT4G02300, AT4G02320, AT4G02330, AT4G03930, AT4G33220, AT4G33230, AT5G04960, AT5G04970, AT5G09760, AT5G20860, AT5G51490, AT5G51500, AT5G53370. PMEI: AT1G02550, AT1G09360, AT1G10770, AT1G23350, AT1G47960, AT1G50340, AT1G62770, AT2G01610, AT2G47050, AT2G47340, AT2G47670, AT3G17130, AT3G17140, AT3G47380, AT3G47670, AT3G62180, AT4G00080, AT4G12390, AT4G25250, AT4G25260, AT5G20740, AT5G38610, AT5G50030, AT5G62350, AT5G64620.

Table S1. RT-qPCR primer targeting *Lepidium sativum* FR14 genes/cDNAs, *L. sativum* reference genes and AGI codes of putative *Arabidopsis thaliana* orthologs.

L. sativum gene/cDNA name	AGI code of putative *A. thaliana* ortholog	Primer name	Primer sequence (5' to 3')
LesaEXPA2	AT5G05290	LesaEXPA2-FP[a]	CAGCCAAGGCTATGGGCTAC
		LesaEXPA2-RP[a]	GCTCACAACAGTCCGACCAT
LesaGAL2	AT5G08370	LesaGAL2-FP3	ATCTACTCTGATGCTGGGACTTT
		LesaGAL2-RP3	GGTATCTCTCCTTCGGACTAGTC
LesaCSLA2	AT5G22740	LesaCSLA2-FP1	GCTCCAATTTCCCAGTAGTTCT
		LesaCSLA2-RP1	ATTCATCAGCATTCACGAAACG
LesaCSLA3	AT1G23480	LesaCSLA3-FP1	TGAGAAGTGGCGTAATTGGAG
		LesaCSLA3-RP1	TGTTGGCGAGTTCAAGATCAT
LesaCESA1	AT4G32410	LesaCESA1-FP1	ACAACCAATCCTGCTACTCTTC
		LesaCESA1-RP1	AGCAAGTCTCTCCAAAAGTCTC
LesaCESA3	AT5G05170	LesaCESA3-FP1	CAGGATTTCAGCACCACAAAAA
		LesaCESA3-RP1	CTTTTCATCGTCAAAACCAGCA
LesaSIP2	AT3G57520	LesaSIP2-FP1	AAGAGTCTCCATGTGTTTCCTG
		LesaSIP2-RP1	AGCCAAACCAGTCTAGGATAGA
LesaDIN10	AT5G20250	LesaDIN10-FP3	CGTATATAACTGCCAAGGAGCA
		LesaDIN10-RP3	GAGTAAACAGCACAGTCTCCAT
LesaUGE2	AT4G23920	LesaUGE2-FP1	GAATTCCCAACAATCTCATGCC
		LesaUGE2-RP1	GCCCAATTCCATAGATCCCTAC
LesaUXS1	AT3G53520	LesaUXS1-FP1	ACAAATGTAATGGGCACTCTCA
		LesaUXS1-RP1	TAGCAACTCCTCTCACCAATTG
LesaRHM3	AT3G14790	LesaRHM3-FP1	GTCTGTTGATGATGAACTTCGC
		LesaRHM3-RP1	CACCACTTTATTGTAACGCGAG
LesaUTR3	AT1G14360	LesaUTR3-FP1	CCACCCAAGATTCCATTACTGT
		LesaUTR3-RP1	ACCAAATACCATCGACACACAT
LesaGAE6	AT3G23820	LesaGAE6-FP1	TATCGTCAACACCGGATACAAG
		LesaGAE6-RP1	GGAAGAGGAAGAGAATAACCCG
LesaXGD1	AT5G33290	LesaXGD1-FP1	AGCAATTAAAAAGGCAGCTTCC
		LesaXGD1-RP1	GTCCTTCAATGCCGTAAATGTC
LesaQUA1	AT3G25140	LesaQUA1-FP1	CTCTTCCTCTTCACTCTCTCCT
		LesaQUA1-RP1	TCCGTTCCTTCACTTCTTTCTC
LesaGATL6	AT4G02130	LesaGATL6-FP1	TCCATCGCTTCAATCATTTCCT
		LesaGATL6-RP1	ACCTTTAACATTGTCACCACCA
LesaXXT1	AT3G62720	LesaXXT1-FP1	GAACTTAAAGACCGACCCGTAT
		LesaXXT1-RP1	CCAAAATTCCCCAATAACCGTG
LesaXXT2	AT4G02500	LesaXXT2-FP1	GGCAAAGCTTCCATTGATTAGG
		LesaXXT2-RP1	TCCACAAGAATTCCCCAGTAAC

… table continued next page

LesaCSLC4	AT3G28180	LesaCSLC4-FP1	GATTAGTCCTCTGTCTCGGTTG
		LesaCSLC4-RP1	TGCTGCTCCTATTGATTGTTCA
LesaBGAL8	AT2G28470	LesaBGAL8-FP1	TTCATTATCCTCGAAGCACTCC
		LesaBGAL8-RP1	ATTCCATTCAGCACAGACGTAA
LesaBGAL10	AT5G63810	LesaBGAL10-FP3	TCAATGGTCTGCTTCTATGGC
		LesaBGAL10-RP3	AACACTTCCACCTTTTCCGAA
LesaXTH8	AT1G11545	LesaXTH8-FP2	CAGTAACACCGGAACAGTCTT
		LesaXTH8-RP2	TCAACGAAGAACACTAGCTGG
LesaXTH9	AT4G03210	LesaXTH9-FP1	TTGACAACTTCTCTGGAGCTG
		LesaXTH9-RP1	TACCATGAATACAACACTGCGT
LesaXTH16	AT3G23730	LesaXTH16-FP2	AACAATGATCGCAACAGCATAC
		LesaXTH16-RP2	TCAGTCTTAACCAAACCACCTC
LesaXTH18	AT4G30280	LesaXTH18-FP1[b]	TGGGGGTTCCATGTGATGCAGT
		LesaXTH18-RP1[b]	GGCTCAGGAGACAAAGAACAACAG
LesaXTH19	AT4G30290	LesaXTH19-FP1[b]	GGGGGCACAGAGAAACTACATGGT
		LesaXTH19-RP1[b]	TTAGCTGCACTCTGGAGGAACACCAC
LesaXTH24	AT4G30270	LesaXTH24-FP1	CCACACTTACTCAATCCTCTGG
		LesaXTH24-RP1	TTCGAGCTACATGACGATCTTC
LesaXTH33	AT1G10550	LesaXTH33-FP1	ATCCGTCAAAACCAATGTCTCT
		LesaXTH33-RP1	TGTGATCTGATTCCTGGACAAC
LesaDOG1a	AT5G45830	LesaDOG1a-FP[a]	CTTTGTGTGGCTCCGAAACT
		LesaDOG1a-FP[a]	GCTCACAACAGTCCGACCAT
Lesa20000 (HQ912757)	AT2G20000	Lesa20000-FP1[c]	TCTGGTCCACGACGGAGCTTG
		Lesa20000-RP1[c]	GCGTTGCTCACATTTCCGCTTACT
Lesa04660 (HQ912754)	AT2G04660	Lesa04660-FP1[c]	AGCTGGGTCTATTGCACGAAGGG
		Lesa04660-RP1[c]	TCGTTTGCTCACTGCTGGTGCTT
Lesa17210 (HQ912755)	AT1G17210	Lesa17210-FP2[c]	TCCGCCCTTGTATGGACGAGAAG
		Lesa17210-RP2[c]	CTCTGGCACCTGGGAAAGCCA

[a] (Graeber *et al.*, 2014), [b] (Voegele *et al.*, 2011), [c] (Graeber *et al.*, 2011)

Graeber K, Linkies A, Steinbrecher T, Mummenhoff K, Tarkowská D, Turečková V, Ignatz M, Sperber K, Voegele A, de Jong H, et al. 2014. *DELAY OF GERMINATION 1* mediates a conserved coat dormancy mechanism for the temperature- and gibberellin-dependent control of seed germination. *PNAS* **111**: E3571-E3580.

Graeber K, Linkies A, Wood AT, Leubner-Metzger G. 2011. A guideline to family-wide comparative state-of-the-art quantitative RT-PCR analysis exemplified with a Brassicaceae cross-species seed germination case study. *The Plant Cell* **23**: 2045-2063.

Voegele A, Linkies A, Müller K, Leubner-Metzger G. 2011. Members of the gibberellin receptor gene family *GID1* (*GIBBERELLIN INSENSITIVE DWARF1*) play distinct roles during *Lepidium sativum* and *Arabidopsis thaliana* seed germination. *Journal of Experimental Botany* **62**(14): 5131-5147.
